# Supplementary material for: Spatial and temporal intra-tumoral heterogeneity in advanced HGSOC: Implications for surgical and clinical outcomes
Source: Cell Rep Med. 2023 May 22;4(6):101055. doi: 10.1016/j.xcrm.2023.101055 (PMC10313917; doi:10.1016/j.xcrm.2023.101055)
Supplement: Document S1. Figures S1–S6 [file mmc1.pdf]

Cell Reports Medicine, Volume *4*

Supplemental information

Spatial and temporal intra-tumoral heterogeneity in advanced HGSOC: Implications for surgical and clinical outcomes

Paula Cunnea, Edward W. Curry, Elizabeth L. Christie, Katherine Nixon, Chun Hei Kwok, Ahwan Pandey, Ratri Wulandari, Kerstin Thol, Jennifer Ploski, Cristina Morera-Albert, Stephen McQuaid, Jingky Lozano-Kuehne, James J. Clark, Jonathan Krell, Euan A. Stronach, Iain A. McNeish, David D.L. Bowtell, and Christina Fotopoulou

Supplementary Information:

Figure S1: Survival analysis for patient cohort related to clinical parameters, clonal evolution, and relapse status.

Figure S2: Heterogeneous clustering patterns of tumor evolution for cases with relapse samples based on inter-deposit CNEvent genomic distances.

Figure S3: Variation in exposure to individual HGSOC copy number signature exposures grouped by relapse status and abdominal location, or clinical parameters.

Figure S4: Survival data for homologous recombination deficiency status, and poor prognosis biomarkers *CCNE1* and *MYC*

Figure S5: Quality control demonstrating copy number of genes associated with poor prognosis, aberrant cell fraction and ploidy for all tumor samples.

Figure S6: Phenotypic apoptosis, cisplatin IC_{50} and proliferative index data related to relapse status and patient outcomes for tumor samples in patient cohort.

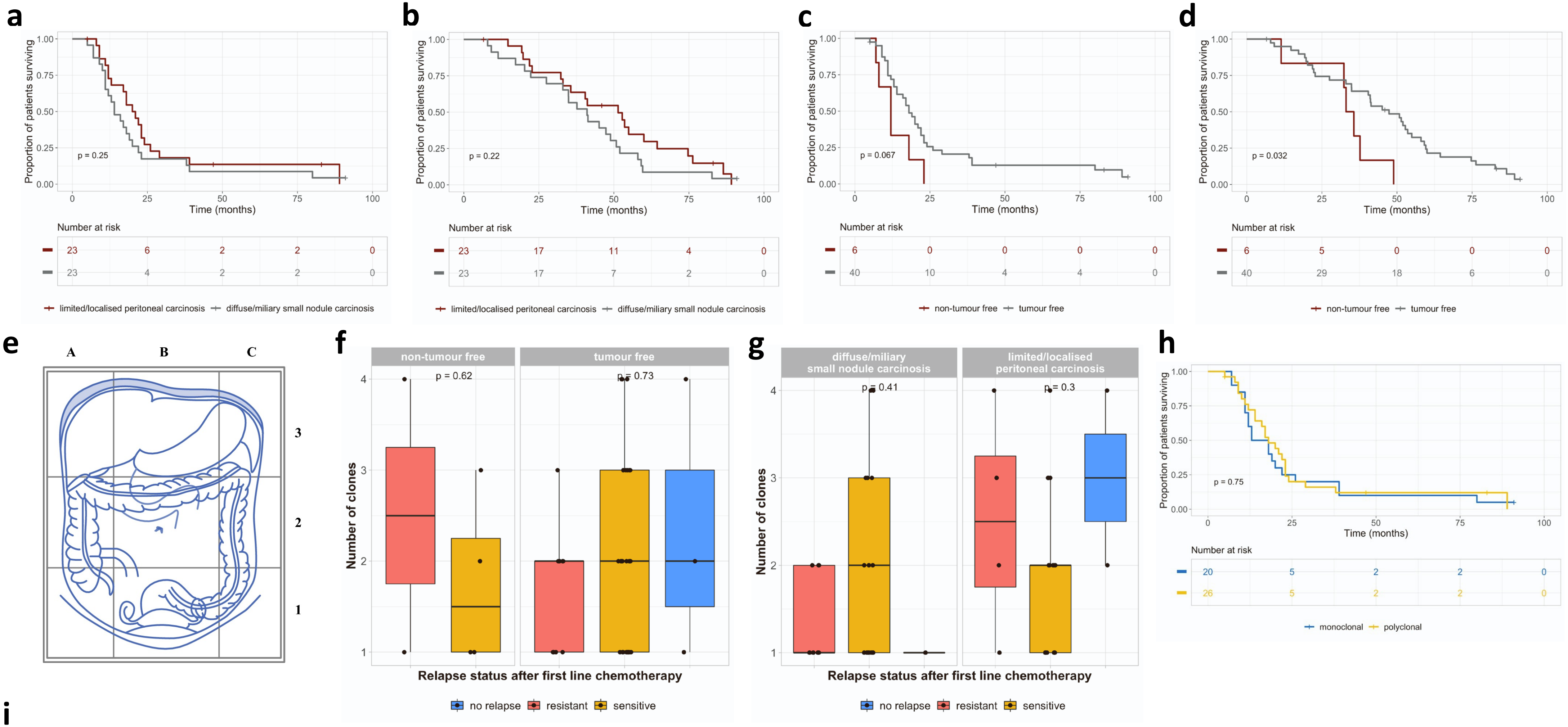

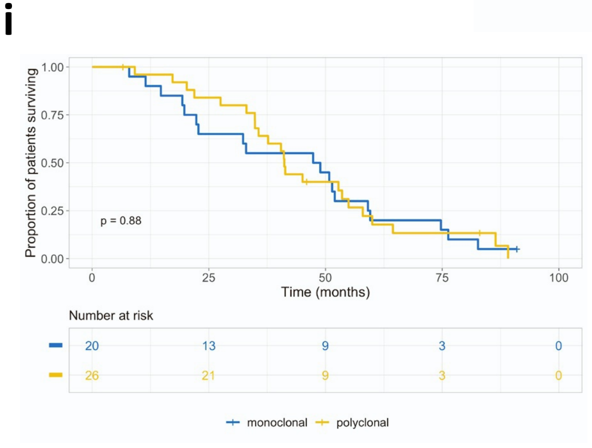

Figure S1 Survival analysis for patient cohort related to clinical parameters, clonal evolution, and relapse status.: a/b) Patient clinical outcomes by clinical presentation. Kaplan-Meier curve showing the association between disease status (limited/localized peritoneal carcinosis; diffuse/military small nodule carcinosis) and progression free survival (**a;** p = 0.25) and overall survival (**b;** p = 0.22) (n = 46). **c/d) Patient clinical outcomes by presence of residual disease following primary debulking surgery (PDS).** Kaplan-Meier curve showing the significant association between tumor-free status (n=40) vs non-tumor free (n=6) and progression free survival (**c;** p = 0.067) and overall survival (**d;** p = 0.032). **e) Intra-operative mapping tool for ovarian cancer (IMO). f) Variation in the number of clones by presence of residual disease post PDS.** Box plot showing the number of clones in primary deposits from patients (n = 46), grouped by presence of residual disease

following PDS (tumor-free versus non-tumor free status) and the relapse status after first line chemotherapy. Wilcoxon signed-rank test was performed across patients of different relapse status within residual disease groups. No statistically significant differences were observed**. g) Variation in the number of clones by tumor dissemination patterns.** Box plot showing the number of clones in primary deposits from patients (n = 46), grouped by the dissemination pattern (limited/localized peritoneal carcinosis; diffuse/military small nodule carcinosis) and relapse status after first line chemotherapy. Wilcoxon signed-rank test was performed across patients of different relapse status within each dissemination pattern group. **h/i) Patient clinical outcome by primary tumor clonal diversity.** Kaplan-Meier plots showing association between number of clones in primary tumor deposits (monoclonal versus polyclonal) and progression-free survival (**c:** PFS, p = 0.75) and overall survival (**d:** OS, p = 0.88). Related to Figure 2.

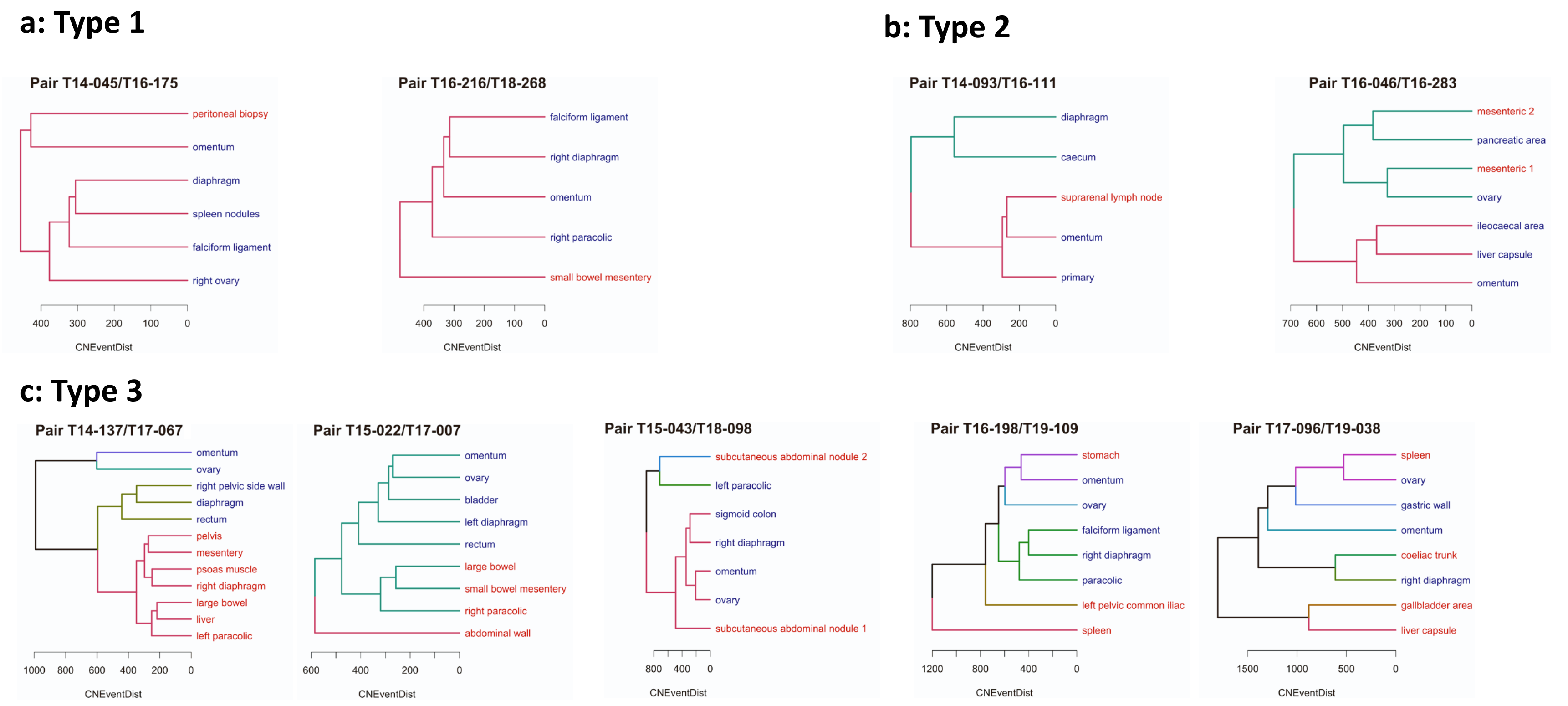

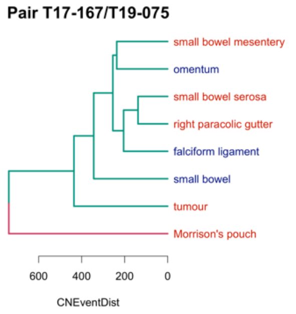

Figure S2: a-c: Heterogeneous clustering patterns of tumor evolution for cases with relapse samples based on inter-deposit CNEvent genomic distances. Companion to Fig.2g, showing the remaining 7 cases with relapse samples categorized into Types 1 (**a**), 2 (**b**) and 3 (**c**) according to the pattern of clonal evolution. Tumors collected at time of primary surgery are labelled in blue, tumors collected at relapse are labelled in red, the color of the dendrogram branches represents different clones.

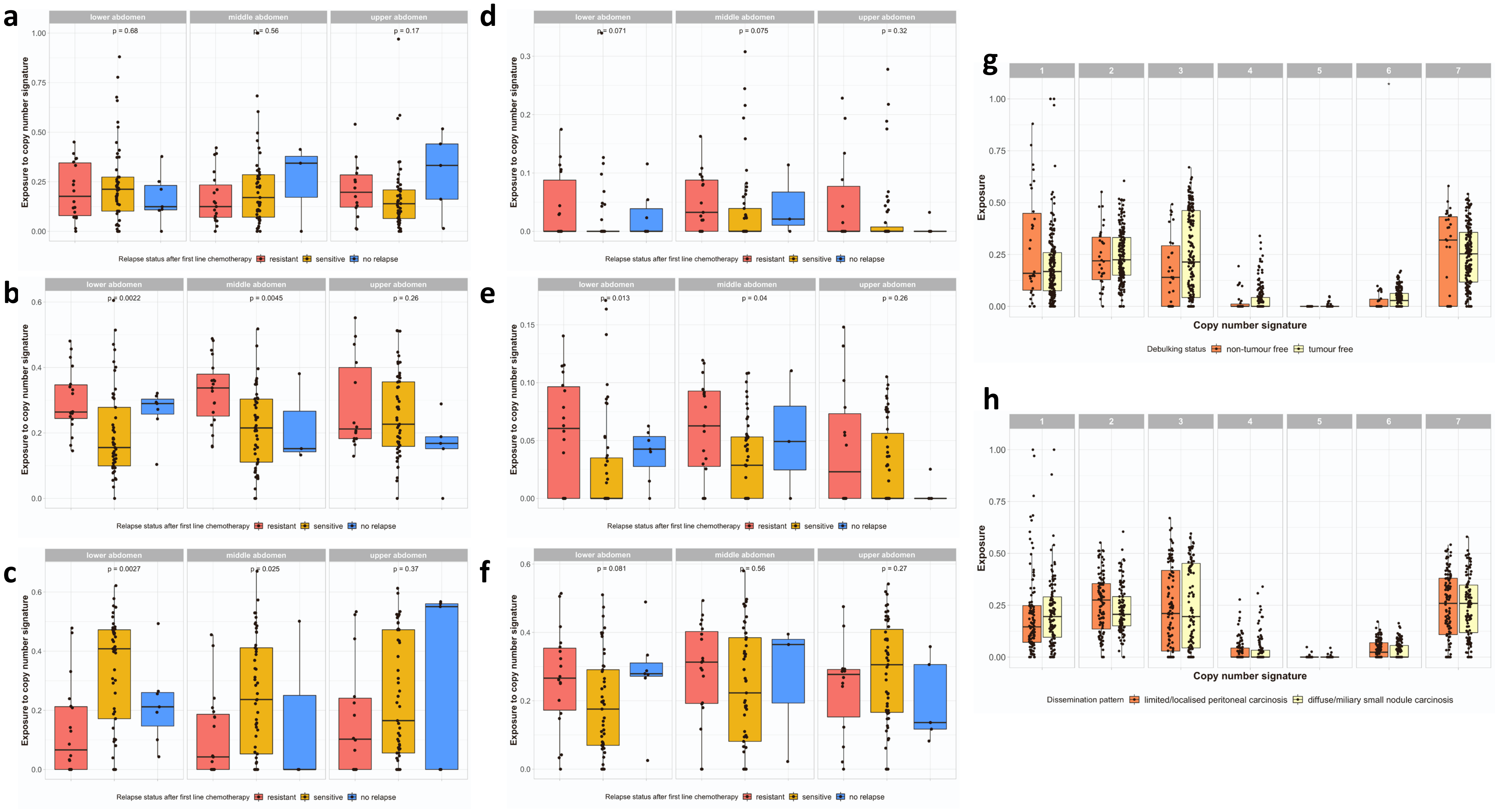

a
lower abdomen
middle abdomen
upper abdomen
p = 0.68
p = 0.56
p = 0.17
Exposure to copy number signature
1.00
0.75
0.50
0.25
0.00
Relapse status after first line chemotherapy
resistant
sensitive
no relapse
b
lower abdomen
middle abdomen
upper abdomen
p = 0.0022
p = 0.0045
p = 0.26
Exposure to copy number signature
0.6
0.4
0.2
0.0
Relapse status after first line chemotherapy
resistant
sensitive
no relapse
c
lower abdomen
middle abdomen
upper abdomen
p = 0.0027
p = 0.025
p = 0.37
Exposure to copy number signature
0.6
0.4
0.2
0.0
Relapse status after first line chemotherapy
resistant
sensitive
no relapse
d
lower abdomen
middle abdomen
upper abdomen
p = 0.071
p = 0.075
p = 0.32
Exposure to copy number signature
0.3
0.2
0.1
0.0
Relapse status after first line chemotherapy
resistant
sensitive
no relapse
e
lower abdomen
middle abdomen
upper abdomen
p = 0.013
p = 0.04
p = 0.26
Exposure to copy number signature
0.15
0.10
0.05
0.00
Relapse status after first line chemotherapy
resistant
sensitive
no relapse
f
lower abdomen
middle abdomen
upper abdomen
p = 0.081
p = 0.56
p = 0.27
Exposure to copy number signature
0.6
0.4
0.2
0.0
Relapse status after first line chemotherapy
resistant
sensitive
no relapse
g
1
2
3
4
5
6
7
Exposure
1.00
0.75
0.50
0.25
0.00
Copy number signature
Debulking status
non-tumour free
tumour free
h
1
2
3
4
5
6
7
Exposure
1.00
0.75
0.50
0.25
0.00
Copy number signature
Dissemination pattern
limited/localised peritoneal carcinosis
diffuse/miliary small nodule carcinosis

Figure S3 Variation in exposure to individual HGSOC copy number signature exposures grouped by relapse status and abdominal location, or clinical parameters: a-f) Variation in exposure to individual HGSOC copy number signature exposures grouped by relapse status and abdominal location (upper (IMO A3-C3), middle (IMO A2-C2) and lower (IMO A1-C1) abdomen). a: Signature 1, **b**: Signature 2, **c**: Signature 3, **d**: Signature 4, **e**: Signature 6, **f**: Signature 7. Statistically significant differences in relapse groups compared to sensitive/no relapse groups were observed in the poor prognosis Signatures 2 and 6 for tumors located in the lower (S2: $p = 0.0022$; S6: $p = 0.013$) and middle (S2: $p = 0.0045$; S6: $p = 0.04$) abdominal areas, but not the upper abdomen (Kruskal-Wallis test). Signature 3 displays significant elevated signature scores in the sensitive/no relapse groups compared to the resistant group in the lower abdomen ($p = 0.027$) and middle abdomen ($p=0.025$). There was little to no representation of Signature 5, related to subclonal catastrophic chromothriptic-like events through unknown mechanisms, in any of the tumors profiled and therefore a graph for signature 5 was not plotted. **g) Variation in exposure to HGSOC copy number signatures grouped by debulking status.** Box plot showing the exposure to 7 copy number signatures of HGSOC for 215 samples from 46 patients, grouped by debulking status of primary surgery (tumor free vs non-tumor free). Wilcoxon signed-rank test was performed across samples from patients of different debulking status within each copy number signature group. Statistical significance was observed for Signature 6 ($p = 0.049$). **h) Variation in exposure to HGSOC copy number signatures by dissemination patterns.** Box plot as in (**e**) grouped by dissemination pattern of tumors observed in patients. Statistical test as (**g**). Related to Figure 3.

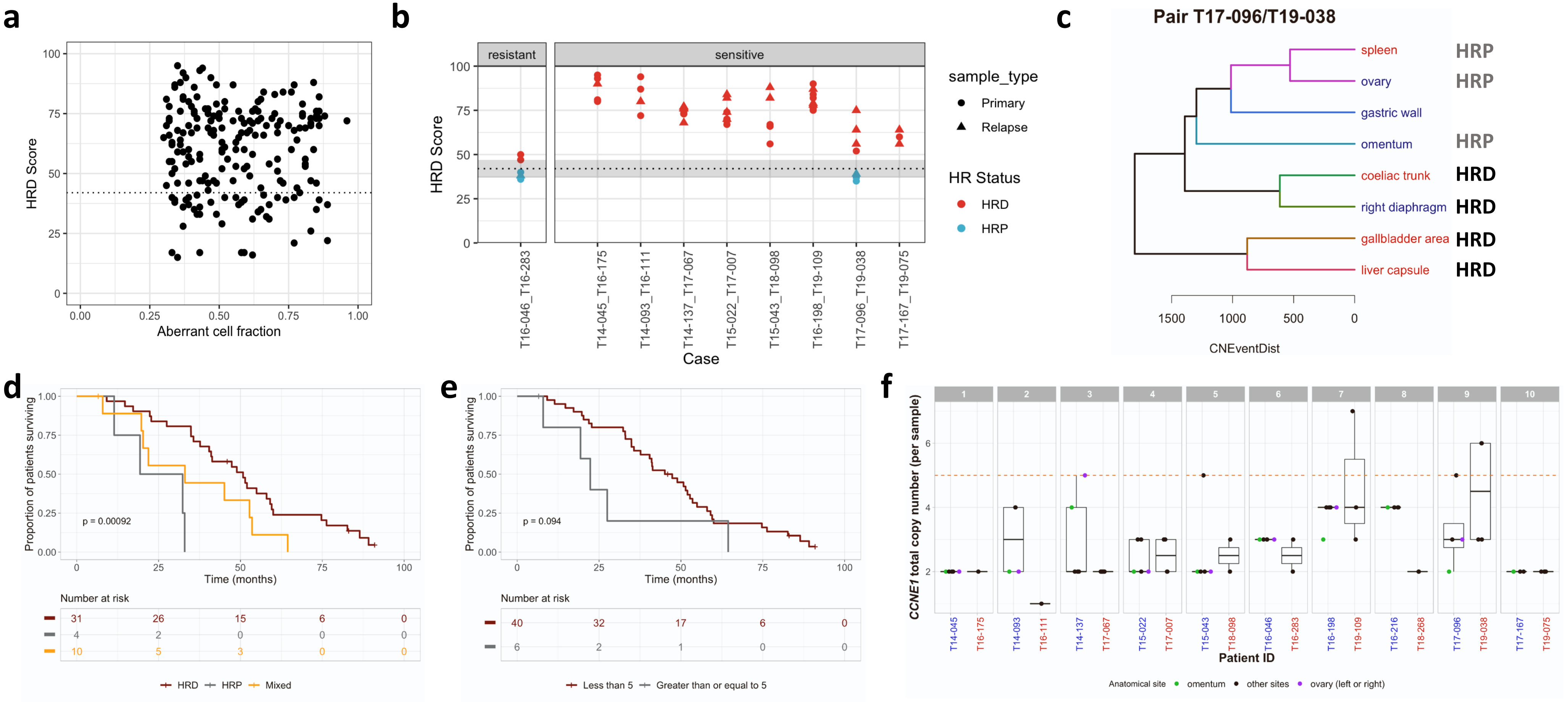

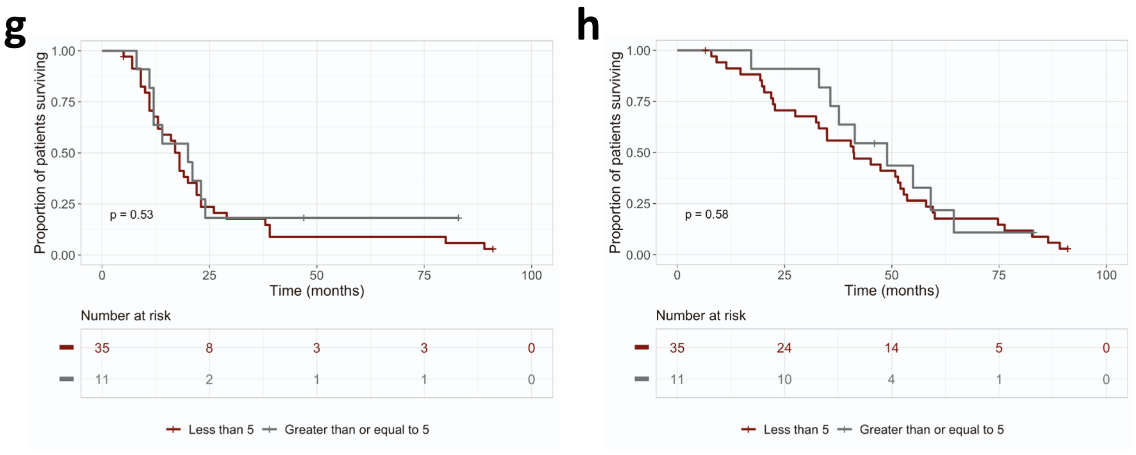

Figure S4 Survival data for homologous recombination deficiency status, and poor prognosis biomarkers CCNE1 and MYC: a) Quality control demonstrating aberrant cell fraction in tumors profiled for HR score. Aberrant cell fraction was estimated for all tumors in the cohort, and those with an ASCAT aberrant cell fraction of <0.3 were excluded due to low tumor purity. Samples also with an ASCAT aberrant cell fraction of 1 and an HRD-LOH, LST and telomeric AI score of 0 were also excluded. Samples were classified as HR deficient (HRD) if the HR Sum score was >42 (dotted line). **b) HR profiles observed in cases with relapse tumor samples.** HR scores are shown for each tumor for 9 patients who had

tumors collected at time of relapse. For two of the patients, mixed HR status was observed within both the primary and relapse samples. **c) Relapse tumors cluster with primary tumors according to HR status.** For patient T17-096, we examined the tumor evolution pattern from primary to relapse as demonstrated by dendrogram derived from tumor genomic distances. The spleen tumor collected at relapse, categorized as HR proficient, clustered with the tumors collected at primary surgery that were also classified as HR proficient. The other relapse samples: coeliac trunk, gall bladder and liver capsule clustered with the diaphragm sample from primary surgery, all classified as HR deficient. **d) Clinical implication of HR status**. Kaplan-Meier curve showing the association between the 3 different HR categories (all tumors within a patient had a HRP profile (HRP), all tumors had a HRD profile (HRD) or if there was a mix of HRP and HRD profiles within a patient's tumors (Mixed)) and overall survival (OS, p = 0.00092). **e) Clinical implication of CCNE1 amplification**. Kaplan-Meier curve showing the association between the mean *CCNE1* total copy number across multiple deposits within a patient and overall survival (OS), split by a mean *CCNE1* copy number of greater or less than 5 copies (OS, p = 0.091). **f) Intra-tumor heterogeneity of *CCNE1* copy number in cases with relapse samples**. Paired box plot showing *CCNE1* total copy number per sample across patients (n = 10), grouped by collection time point and points colored by tumor anatomical site. Blue text indicates tumor deposits collected from the primary surgery, while red text indicates tumor deposits collected at relapse. **g/h) Clinical association of *MYC* amplification**. Kaplan-Meier curve showing the association between the mean *MYC* total copy number across multiple deposits within a patient and progression free survival (**g:** PFS, p = 0.53) and overall survival (**h:** OS, p = 0.58), with patients stratified into 2 groups based on the mean *MYC* copy-gain of less than or great than or equal to 5 copies. Related to Figure 4.

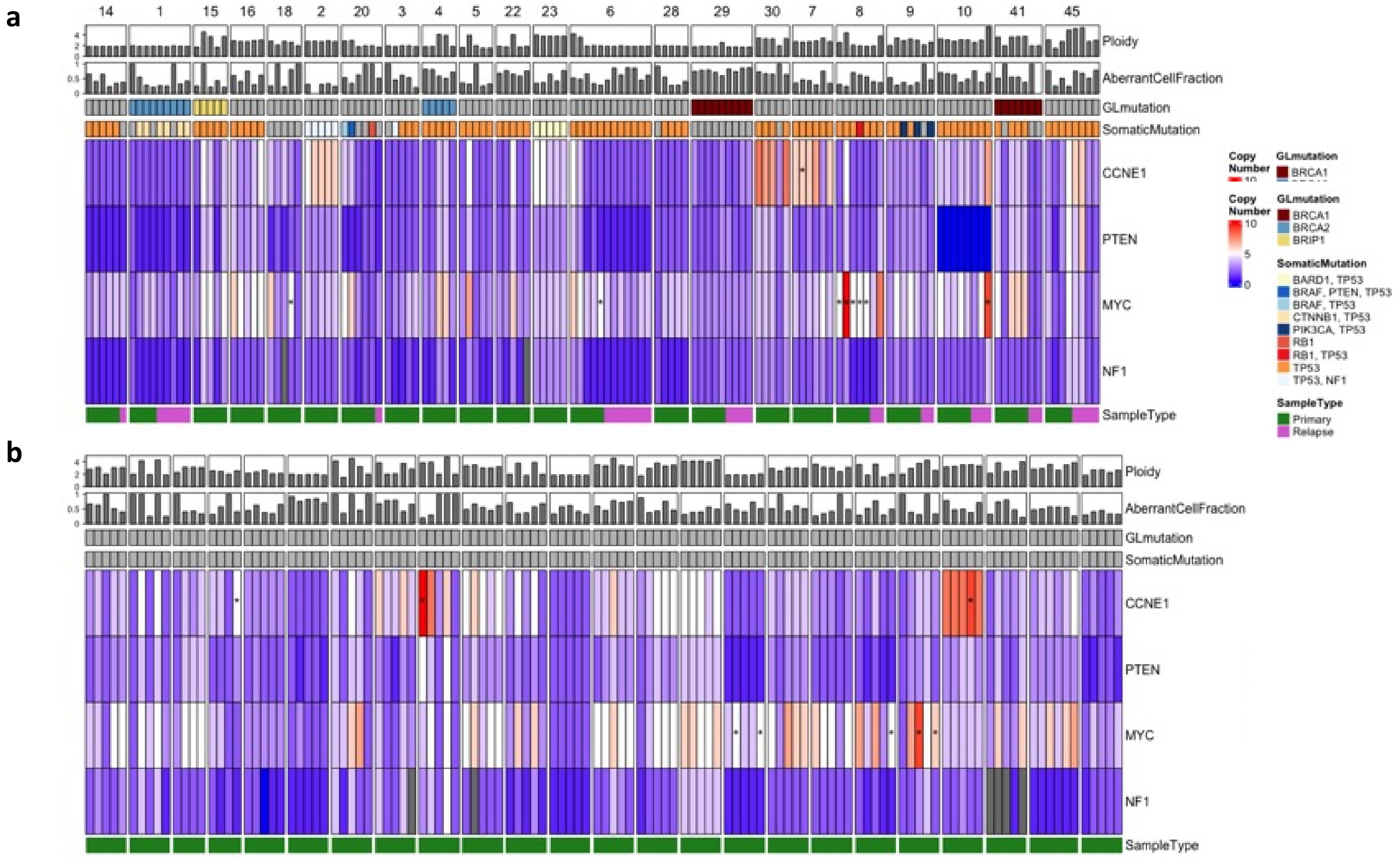

Figure S5: Quality control demonstrating copy number of genes associated with poor prognosis, aberrant cell fraction and ploidy for all tumor samples. a) Heatmap depicting the *CCNE1, MYC, PTEN* and *NF1* total CN in all tumors profiled (n=259), annotated by tumor ploidy, aberrant cell fraction, and sample collection time. Samples from the same case are grouped together. Gene amplification as defined by COSMIC (ploidy ≤2.7 and total CN ≥5 or ploidy >2.7 and total CN ≥9) is highlighted using asterisks*. Somatic and germline mutational data available for 21 patients including matched relapse is displayed according to data legend. **b)** Heatmap as in **(a)** depicting *CCNE1, MYC, PTEN* and *NF1* total CN in tumors, without germline or somatic mutational data. See also Figure 4d; Supplementary File 1 - Table S1f.

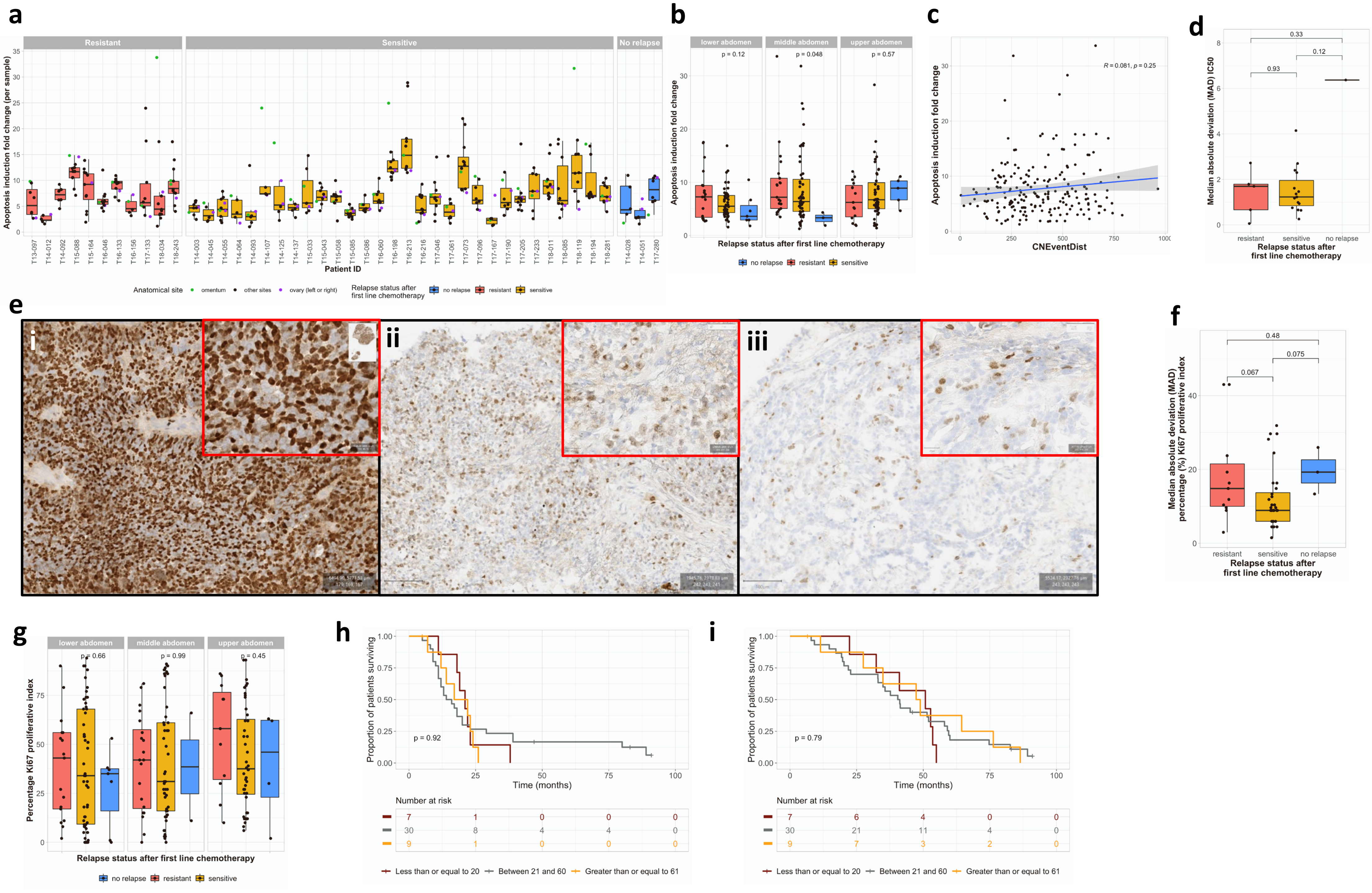
a
Resistant
Sensitive
No relapse
Apoptosis induction fold change (per sample)
Patient ID
Anatomical site
omentum
other sites
ovary (left or right)
Relapse status after first line chemotherapy
no relapse
resistant
sensitive
b
lower abdomen
middle abdomen
upper abdomen
p = 0.12
p = 0.048
p = 0.57
Apoptosis induction fold change
Relapse status after first line chemotherapy
no relapse
resistant
sensitive
c
Apoptosis induction fold change
R = 0.081, p = 0.25
CNEventDist
d
Median absolute deviation (MAD) IC50
0.33
0.12
0.93
resistant
sensitive
no relapse
Relapse status after first line chemotherapy
e
i
ii
iii
f
Median absolute deviation (MAD) percentage (%) Ki67 proliferative index
0.48
0.075
0.067
resistant
sensitive
no relapse
Relapse status after first line chemotherapy
g
lower abdomen
middle abdomen
upper abdomen
p = 0.66
p = 0.99
p = 0.45
Percentage Ki67 proliferative index
Relapse status after first line chemotherapy
no relapse
resistant
sensitive
h
Proportion of patients surviving
p = 0.92
Time (months)
Number at risk
7 1 0 0 0
30 8 4 4 0
9 1 0 0 0
Less than or equal to 20
Between 21 and 60
Greater than or equal to 61
i
Proportion of patients surviving
p = 0.79
Time (months)
Number at risk
7 6 4 0 0
30 21 11 4 0
9 7 3 2 0
Less than or equal to 20
Between 21 and 60
Greater than or equal to 61

Figure S6 Phenotypic apoptosis, cisplatin IC50 and proliferative index data related to relapse status and patient outcomes for tumor samples in patient cohort: a) *In vitro* apoptosis levels following platinum chemotherapy treatment by patient. Related to Figure 5. Box plot of phenotypic apoptosis induction of tumor cultures derived from each patient according to relapse status. Primary 2D-tumor cell cultures were established from disseminated tumors (mean=9, range 4-15) and treated in triplicate with cisplatin to evaluate *in vitro* apoptotic responses to platinum chemotherapy, data was normalized to viability and a fold change of apoptosis induction was determined compared to vehicle control for each tumor, n=1 technical replicates. **b) *In vitro* apoptosis levels following platinum chemotherapy treatment by abdominal location.** Functional heterogeneity of primary tumor cells is demonstrated using a box plot showing the apoptosis induction fold change of primary cell cultures that were extracted and grown from tumor deposits by abdominal location (lower, middle, or upper), grouped by relapse status. There is a trend towards higher apoptosis induction in the resistant group in the lower ($p = 0.12$) and middle ($p = 0.048$) abdominal areas (Kruskal-Wallis test). **c) Association of apoptotic response with genomic distance.** Non-significant correlation between genomic distance (CNEventDist) and apoptotic induction for each tumor per patient (r=0.081, p=0.25). **d) Functional heterogeneity of primary tumor cells.** Box plot showing the median absolute deviation (MAD) of sample-wise IC50 for cisplatin from primary cell cultures that were grown from tumor deposits from a subset of patients (n=21), grouped by relapse status. Kruskal-Wallis test, p=0.2537, related to Table S1i. **e) Percentage Ki67 proliferative index examples of staining categories.** Related to Figure 5e. Representative images of each of the categories: **i** High Ki67, **ii** Moderate Ki67, **iii** Low Ki67. Main images are at x4 magnification and inserts at x20 magnification. Scale bar at 100 µm. **f) Heterogeneity in Ki67 proliferative index of primary tumors.** Box plot showing the median absolute deviation (MAD) of sample-wise Ki67 proliferative index of tumor deposits from patients (n = 46 patients), grouped by relapse status (Kruskal-Wallis test, p = 0.05278). **g) Distribution of Ki67 proliferative index scores across different abdominal areas.** Percentage Ki67 proliferative index was grouped by relapse status and plotted according to abdominal location (lower, middle, upper). No statistically significant differences were observed across the 3 abdominal areas and relapse groups (Kruskal-Wallis test). **h/i) Clinical association of Ki67 proliferative index**. Kaplan-Meier curve showing the association between the mean Ki67 PI across multiple deposits within a patient and progression free survival (PFS, p = 0.92) and overall survival (OS, p = 0.79), with positive Ki67 proliferative index staining stratified into 3 categories: low (<20%), moderate (21-60%) and high (>60%) across the cohort (n=46)
