# Supplementary material for: Spatial and temporal intra-tumoral heterogeneity in advanced HGSOC: Implications for surgical and clinical outcomes
Source: Cell Rep Med. 2023 May 22;4(6):101055. doi: 10.1016/j.xcrm.2023.101055 (PMC10313917; doi:10.1016/j.xcrm.2023.101055)
Supplement: Document S2. Article plus supplemental information [file mmc3.pdf]

Cell Reports
Medicine

Article

Spatial and temporal intra-tumoral heterogeneity in advanced HGSOC: Implications for surgical and clinical outcomes

Graphical abstract

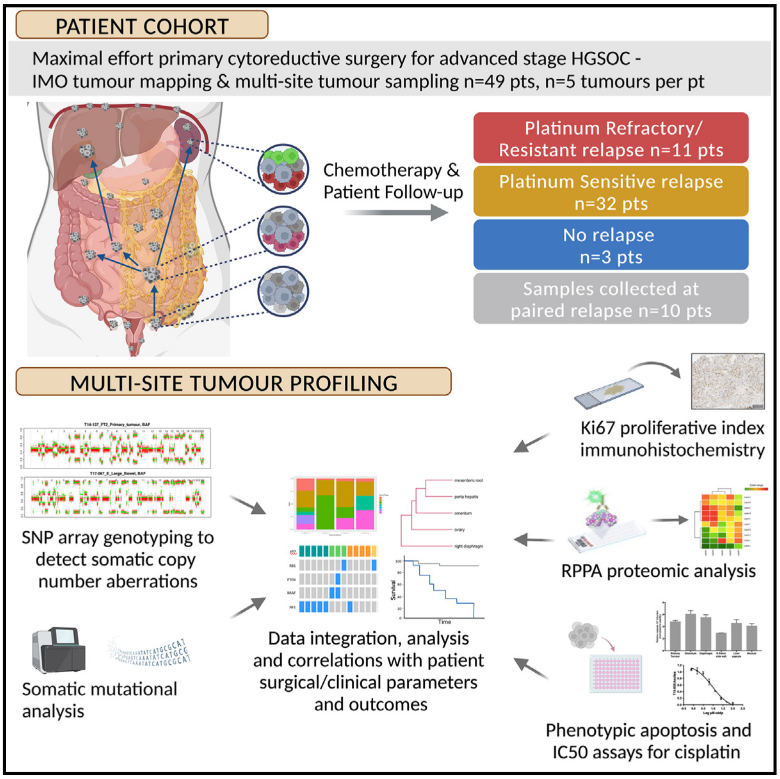

Authors

Paula Cunnea, Edward W. Curry, Elizabeth L. Christie, ..., Iain A. McNeish, David D.L. Bowtell, Christina Fotopoulou

Correspondence

p.cunnea@imperial.ac.uk (P.C.),
c.fotopoulou@imperial.ac.uk (C.F.)

In brief

Cunnea et al. profile a cohort of advanced, high-tumor-burden high-grade serous ovarian cancer patients using systematic, multi-site, multi-level mapping to demonstrate tumor evolution from initial diagnosis through to matched relapse. They report distinct patterns of tumor evolution and extensive spatial and temporal heterogeneity in actionable genomic biomarkers.

Highlights

- Multi-site tumor profiling of high-disease-burden HGSOC patients across space and time
- Distinct tumor evolution patterns identified from initial diagnosis to relapse
- HRD scores and genomic biomarkers demonstrate spatial and temporal heterogeneity
- No adverse tumor profiles identified that preclude patients from cytoreductive effort

Cunnea et al., 2023, Cell Reports Medicine *4*, 101055
June 20, 2023 © 2023 The Authors.
https://doi.org/10.1016/j.xcrm.2023.101055

CellPress

Cell Reports Medicine

CellPress
OPEN ACCESS

Article

Spatial and temporal intra-tumoral heterogeneity in advanced HGSOC: Implications for surgical and clinical outcomes

Paula Cunnea,[1,7,*] Edward W. Curry,[1] Elizabeth L. Christie,[2,3] Katherine Nixon,[1] Chun Hei Kwok,[1] Ahwan Pandey,[2] Ratri Wulandari,[1] Kerstin Thol,[1] Jennifer Ploski,[1] Cristina Morera-Albert,[1] Stephen McQuaid,[4] Jingky Lozano-Kuehne,[5] James J. Clark,[1] Jonathan Krell,[1] Euan A. Stronach,[1] Iain A. McNeish,[1] David D.L. Bowtell,[2,3] and Christina Fotopoulou[1,6,*]

[1]Division of Cancer, Department of Surgery and Cancer, Imperial College London, London W12 0NN, UK

[2]Peter MacCallum Cancer Centre, Melbourne, VIC 3000, Australia

[3]The Sir Peter MacCallum Department of Oncology, The University of Melbourne, Melbourne, VIC 3010, Australia

[4]Queen's University Belfast, Belfast BT7 1NN, UK

[5]Experimental Cancer Medicine Centre, Department of Surgery and Cancer, Imperial College London, London W12 0NN, UK

[6]West London Gynaecological Cancer Centre, Imperial College NHS Trust, London W12 0HS, UK

[7]Lead contact

*Correspondence: p.cunnea@imperial.ac.uk (P.C.), c.fotopoulou@imperial.ac.uk (C.F.)

https://doi.org/10.1016/j.xcrm.2023.101055

SUMMARY

Limited evidence exists on the impact of spatial and temporal heterogeneity of high-grade serous ovarian cancer (HGSOC) on tumor evolution, clinical outcomes, and surgical operability. We perform systematic multi-site tumor mapping at presentation and matched relapse from 49 high-tumor-burden patients, operated up front. From SNP array-derived copy-number data, we categorize dendrograms representing tumor clonal evolution as sympodial or dichotomous, noting most chemo-resistant patients favor simpler sympodial evolution. Three distinct tumor evolutionary patterns from primary to relapse are identified, demonstrating recurrent disease may emerge from pre-existing or newly detected clones. Crucially, we identify spatial heterogeneity for clinically actionable homologous recombination deficiency scores and for poor prognosis biomarkers *CCNE1* and *MYC*. Copy-number signature, phenotypic, proteomic, and proliferative-index heterogeneity further highlight HGSOC complexity. This study explores HGSOC evolution and dissemination across space and time, its impact on optimal surgical cytoreductive effort and clinical outcomes, and its consequences for clinical decision-making.

INTRODUCTION

There have been significant advances in the management of high-grade serous ovarian cancer (HGSOC) in recent years at both surgical and systemic treatment levels.[1] Although cure rates are relatively unchanged, both progression-free (PFS) and overall survival (OS) of patients are improving through the intensification of surgical effort,[2,3] the addition of targeted agents and anti-angiogenic agents to traditional cytotoxic regimens also within maintenance concepts,[4] and the implementation of a more holistic package of care that addresses the patients' needs in an individualized manner.[5] Originating in the fallopian tube in many cases,[6] nearly 80% of HGSOC patients have already widely disseminated peritoneal disease at initial presentation, necessitating complex multivisceral resection techniques to achieve complete macroscopic tumor clearance.[2,7]

Paralleling therapeutic advances, our understanding of the genomic landscape driving HGSOC has equally expanded. Near-ubiquitous *TP53* loss-of-function mutations,[8] defects in homologous recombination (HR) repair, and extensive copy-number (CN) aberrations (CNAs) are features of genomic heterogeneity of HGSOC.[9–11] Approximately 25% of HGSOC patients have a germline, somatic, or epigenetic alteration in *BRCA1* and *BRCA2.* Further common genomic aberrations include inactivation of tumor suppressor genes *PTEN*, *RB1*, *NF1*, and *RAD51B* by gene breakage,[10,12] as well as amplifications and mutations in cell-cycle-mediating genes *CCNE1* and *CDK12*.[12]

Previous attempts to describe spatial heterogeneity of HGSOC at genomic, immunological, and proteomic levels through multisite tumor sampling studies have involved only a limited number of patients and/or intraabdominal tumor sites collected in a nonsystematic pattern.[13–19] Moreover, evidence describing temporal heterogeneity reflecting a patient's treatment journey over time is minimal.[12,18,20–22] Due to the various technical and logistical difficulties in accessing sufficient matched multifocal tumor samples from disseminated primary and recurrent disease, mapping the evolution of advanced HGSOC from primary to relapse remains elusive. As a result, most attempts to identify reliable prognostic and predictive molecular signatures for surgical and clinical outcomes in HGSOC have been somewhat unproductive,[18,21,23–26] with BRCA and HR status still the only predictive molecular biomarkers in regular clinical use.[27,28]

This is an open access article under the CC BY license (http://creativecommons.org/licenses/by/4.0/).

Table 1. Table summarizing the patient demographics, surgical procedures, and tumor-related characteristics, as well as survival data of study cohort

Summary of patient clinical characteristics	n	%
No. of patients	49	
Age at diagnosis (years)		
Mean	62	–
Range	32–91	–
FIGO tumor stage		
III	28	57.1
IV	21	42.9
Residual disease		
Tumor free (non-visible)	42	85.7
Non-tumor free (any visible disease)	7	14.3
Surgical procedures		
Bowel resection	38	77.6
Splenectomy	22	44.9
Bulky LN resection	25	51
Diaphragmatic and/or liver capsule resection	39	79.6
Pleurectomy and/or paracardiac LN removal	11	22.5
Disease dissemination		
Diffuse/miliary small-nodule carcinosis	24	49
Limited/localized peritoneal carcinosis	25	51
Clinical BRCA status		
Wild-type	20	40.8
BRCA1/2 mutant	9	18.4
Not tested	20	40.8
First-line chemotherapy		
Completed first-line chemotherapy	46	93.9
Carboplatin	8	17.4
Carboplatin and paclitaxel	21	45.6
Carboplatin and paclitaxel and bevacizumab	16	34.7
Carboplatin and paclitaxel and avelumab	1	2.2
Relapse status following first-line chemotherapy		
Refractory/resistant	11	22.5
Sensitive	32	65.3
No relapse	3	6.1
Death not due to disease progression	3	6.1
Progression-free survival[a] (months)		
Median	17.5	–
IQR	11–23	–
Overall survival[a] (months)		
Median	41.3	–
IQR	23.9–57.2	–
Follow-up[a] (months)		
Median	74.7	–
IQR	53.6–86.4	–
Deceased during follow-up[a]		
Yes	31	67.4
No	15	32.6
Samples collected at PDS		
Mean per patient	9	–
Range	4–15	–
Samples collected at relapse		
Mean per patient	3	–
Range	1–7	–

IQR, interquartile range; LN, lymph node; PDS, primary debulking surgery.

[a]Data exclude those three patients whose death was not due to disease progression.

There are no valid comprehensive data so far to decode the association of spatial and temporal tumor heterogeneity with tumor dissemination patterns, operability, response to platinum chemotherapy, patterns of relapse, PFS, and OS in advanced HGSOC patients. In order to achieve that in an unbiased way, we set out to evaluate tumor samples from HGSOC patients undergoing primary cytoreductive surgery, before any chemotherapy-induced alterations. We focused on the systematic mapping of tumor dissemination patterns, based on validated structured collection algorithms for ovarian neoplasms,[29,30] eliminating any bias that would potentially derive from arbitrary tumor harvesting. Also, because the same team operated in both the primary and relapsed setting, direct correlations could be made between primary and relapsed tumor dissemination patterns that also guided targeted tumor harvesting. Genomic, proteomic, phenotypic, and anatomical spatial and temporal heterogeneity features of the samples were correlated with surgical and clinical outcomes with the aim of identifying molecular signatures associated with less favorable outcomes despite optimal patient treatment.

RESULTS

Patient cohort and study design

Between September 2013 and November 2018, we recruited 49 patients who underwent primary maximal effort cytoreductive surgery in a center of excellence for ovarian cancer surgery,[31] with systematic banking of tumor samples. Patient demographics, surgical procedures, and tumor-related characteristics, as well as survival data, are summarized in Table 1. Forty-two patients (85.7%) were macroscopically tumor free following surgery, and 46/49 (93.9%) received postoperative chemotherapy. Three patients died not as a result of disease progression and were excluded from further analysis. Within a median follow-up period of 74.7 months (interquartile range [IQR], 53.6–86.4), 43/46 patients (93.5%) experienced disease relapse, and 31/46 patients (67.4%) died during follow-up. Nine of the patients underwent debulking surgery at relapse, and one patient had a biopsy at relapse. Patients were categorized into three

groups (resistant, sensitive, and no relapse) according to their PFS from the end of first-line chemotherapy to their first relapse (see STAR Methods).

Intraoperative tumor dissemination patterns and tumor burden were systematically documented for each patient using the Intraoperative Mapping of Ovarian Cancer (IMO) system (Figure S1E).[29] Most patients had a high tumor burden, with tumor dissemination in all abdominal quadrants, allowing for collection of biopsies from multiple sites. Patterns of peritoneal carcinosis were documented and specified as diffuse/miliary small nodule carcinosis (49%) or limited/localized peritoneal carcinosis (51%).[30,32] A mean of nine tumor samples per patient (range 4–15) were collected from primary surgery (Table S1A). A mean of three relapsed tumor sites were sampled (range 1–7) at relapse by cytoreductive surgery or biopsy.

No association was observed for the different patterns of carcinosis with PFS or OS (Figures S1A and S1B). Patients without macroscopic disease following primary surgery had a significantly longer OS (median OS, 45.5 months; IQR, 22.6–59.2 months) than those patients with residual postoperative disease (median OS, 35.4 months; IQR, 32.5–37.2) (p = 0.032) and longer PFS (p = 0.067; Figures S1C and S1D).

Distinct patterns of genomic heterogeneity and tumor evolution in primary and relapsed HGSOC

An outline of the study structure and genomic analysis workflow is shown in Figure 1. Genome-wide SNP arrays were used to measure genomic CN alterations from primary tumor and germline DNA samples for 49 patients, with matched relapse tumors where available. This approach was taken because somatic genetic alterations in HGSOC predominantly feature CN alterations,[33] and SNP array data remain effective in characterizing such changes.[34]

To quantify the genomic divergence of a pair of tumor samples, we estimated the number of CN alteration events that would need to occur to make the two samples' profiles identical (Figure 2A; STAR Methods) and referenced each tumor sample to its matched germline DNA. This approach is a version of the unweighted CND benchmarked in Zeira and Raphael.[35] Figure 2B shows the tumor genomic distances for each patient, according to their response to treatment. The extent of CN change varied across patients but without a clear association to patient outcome. Pairwise genomic divergence was also estimated for each tumor sample within a patient to produce a matrix of intra-tumoral genomic distances. Hierarchical clustering of genomic distance matrices provided a dendrogram, as an approximation of the clonal evolution of each patient's disease, as in a previous study.[18] Figure 2C illustrates the resulting dendrogram for one patient, showing two distinct branches each containing two tumors with similar genomic CN profiles. By comparing the distributions of genomic distances between pairs of samples from the same patient with pairs of samples from different patients, we identified a threshold for calling distinct clones within each patient (Figure 2D). We did not observe any statistically significant associations between numbers of clones with PFS, OS, treatment response (resistant, sensitive, or no-relapse), residual disease, or carcinosis pattern (Figure 2E; Figures S1F–S1I).

Each patient's dendrogram, representing tumor evolution, was classified using branching terms "sympodial" or "dichotomous." The sympodial pattern describes cases where one branch ceases to diverge at each branching point (Figure 2F; T17-046), whereas dichotomous depicts cases where each branch diverges into two equal branches at each branching point (Figure 2F; T18-011). Different tumor clones derived from genomic distances are represented by a different color branch in each dendrogram, based on the threshold determined for calling a distinct clone. Heterogeneous clustering patterns of tumor evolution were observed in the sensitive group, whereas most resistant patients demonstrated a sympodial structure, and non-relapsed patients had only the dichotomous clustering pattern (p = 0.03525; Figure 2F, lower panel).

There were three distinct patterns of tumor evolution in the 10 patients who had paired tumors collected at both primary and relapse: type 1 (Figure 2G, upper left panel) describes patients in whom only a single clone was detected in all primary (blue text) and relapse (red text) tumors profiled. Type 2 (upper middle panel) demonstrates polyclonality in the primary samples, and the paired relapse samples originate from one of these clones. In type 3 cases, at least one of the relapse tumors comprises a distinct clone that was not detected in the primary disseminated tumor sites (upper right of panel). Most cases (60%) display type 3 topology (Figure 2G, lower panel; Figure S2). Due to the small number of cases with paired relapse tumors as a result of mostly operating on platinum-sensitive patients as per clinical guidelines and selection algorithms, no significant association with outcome could be derived.

CN signature exposures of disseminated HGSOC reveals extensive genomic heterogeneity

Previously we reported seven different CN signatures for HGSOC, predictive of OS,[21] and we applied these methods to our cohort. To demonstrate the range of observed intra-tumoral heterogeneity in CN signatures, we present CN profiles from two example cases exhibiting different exposure scores for each signature (Figure 3A; Table S1E). Although all five tumor samples from patient T18-085 display similar levels of each of several signatures, heterogeneity is observed in patient T16-046. Specifically, signature 4 is present only within the ileocecal, liver capsule, and omentum tumors, whereas signature 6 exposure is present in all tumors except the ovary tumor. Signature 3 is the predominant signature in the ovary tumor, with little signature 7 present. To examine the degree of variation in CN signatures across a patient's disease, we computed the mean of the sum-squared differences in CN signature scores between all pairs of samples from each patient. Patients exhibited variations in mean inter-deposit CN signature distances across the different response categories but without any significant association between heterogeneity and outcome (Figure 3B).

Examining each individual CN signature exposure across the cohort, tumors from the resistant group showed increased exposure scores for signatures 2 (p = 0.00017), 4 (p = 0.0029), and 6 (p = 0.001), related to poor survival (Figure 3C).[21] There was very low representation of signature 5 overall. Increased exposure scores were detected for signature 3 (p = 0.00018), related to *BRCA1/2*-related HRD and favorable survival, within the

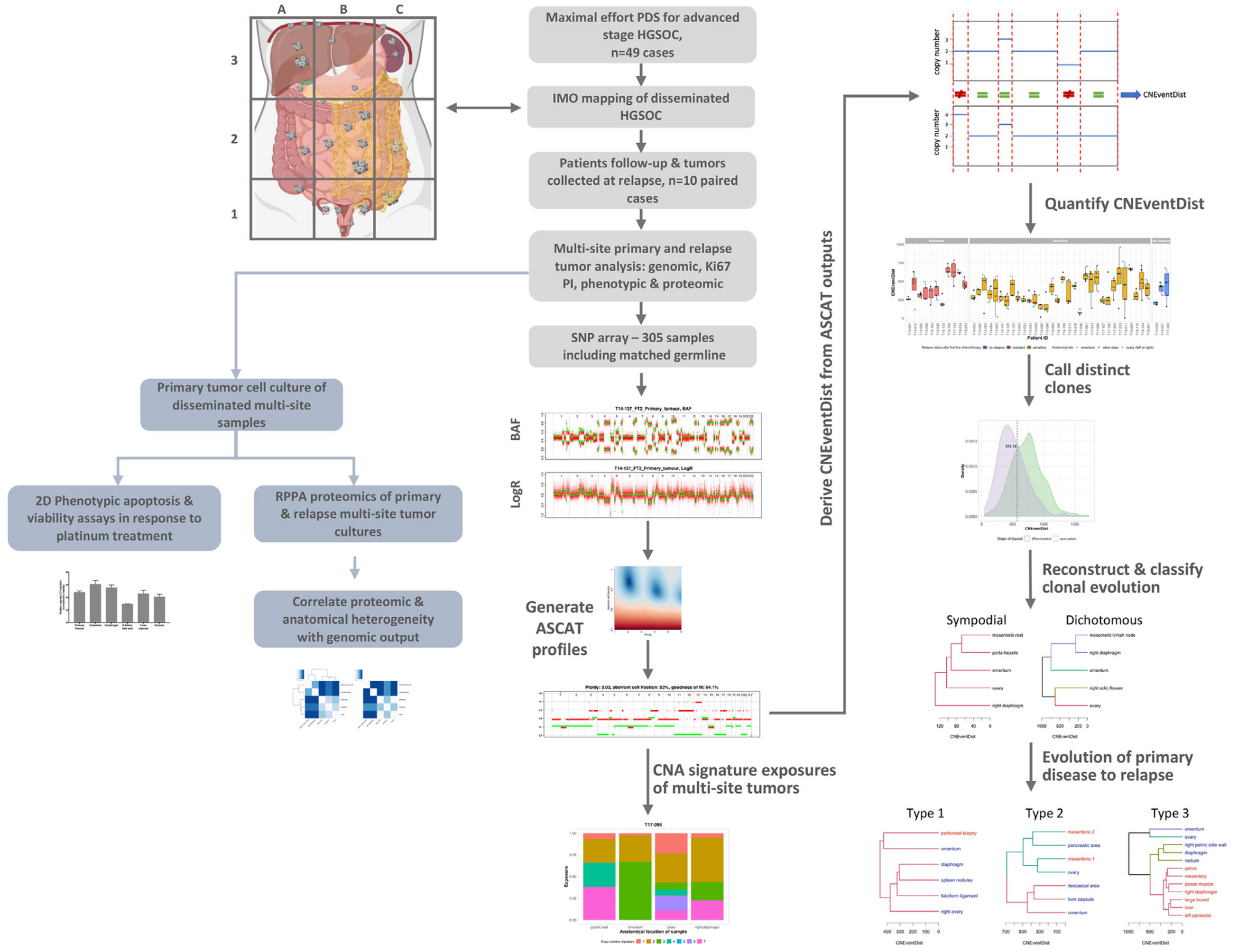

Figure 1. Study outline

Flow diagram outlining the study cohort, genomic procedures, and analysis performed on all samples. The cohort comprised HGSOC patients with advanced disease, high tumor burden, and carcinosis disseminated throughout the entire peritoneal cavity and in some cases in the paracardiac and pleural cavities. Tumors were collected from the entire peritoneal and extraperitoneal cavity, such as the upper abdomen (spleen, lesser sac, celiac trunk, diaphragm/Morison's pouch, liver capsule, retroperitoneal pelvic, and paraortic lymph nodes), the bowel, mesentery, parietal and visceral peritoneum, and pleura and paracardiac lymph nodes, where present. Each tumor sample collected was split for nucleotide extraction for subsequent genomic analysis and primary tumor cell culture. SNP array genotyping was performed on 305 primary and relapsed tumor samples and matched germline samples. These data were used to generate genome-wide allele-specific CN profiles, which in turn were used to reconstruct the clonal evolution of disease for each patient in the cohort. Patterns of clonal evolution were catalogued across the cohort, and associations between genomic heterogeneity and phenotypes, proteomic profiles, and anatomical heterogeneity were explored. CNA, copy-number aberration; CNEventDist, copy-number aberration event distance; PDS, primary debulking surgery; RPPA, reverse phase protein array.

sensitive and no-relapse groups. No significant differences were observed when correlating individual signature exposures with residual disease or carcinosis patterns (Figures S3G and S3H). When examining signature exposure scores according to abdominal location of tumors, upper (IMO location A3, B3, and C3), mid (A2, B2, and C2), and lower abdomen (A1, B1, and C1), tumors from the lower and middle abdomen followed the same pattern as the overall cohort with increased exposure scores for signatures 2, 4, and 6 in the resistant group (Figures S3B, S3D, and S3E) and increased signature 3 in the sensitive and no-relapse groups (Figure S3C). In the upper abdomen, there were no significant differences between poor prognosis signatures and relapse groups (Figures S3A, S3B, and S3D–S3F).

Derived CN signature exposures reflect underlying mutational processes. Therefore, we examined whether CN signature exposures score heterogeneity was related to genomic heterogeneity as determined from the genomic distances for each patient. For all patients with genomic distances and CN signatures derived for five tumors (n = 30), a positive correlation was demonstrated (r = 0.341, p = 0.0653; Figure 3D). CN signature exposures were also calculated for all patients with relapse tumors and examined alongside tumor evolution based on genomic distances (Figure 3E). For most cases, heterogeneity in the CN signature

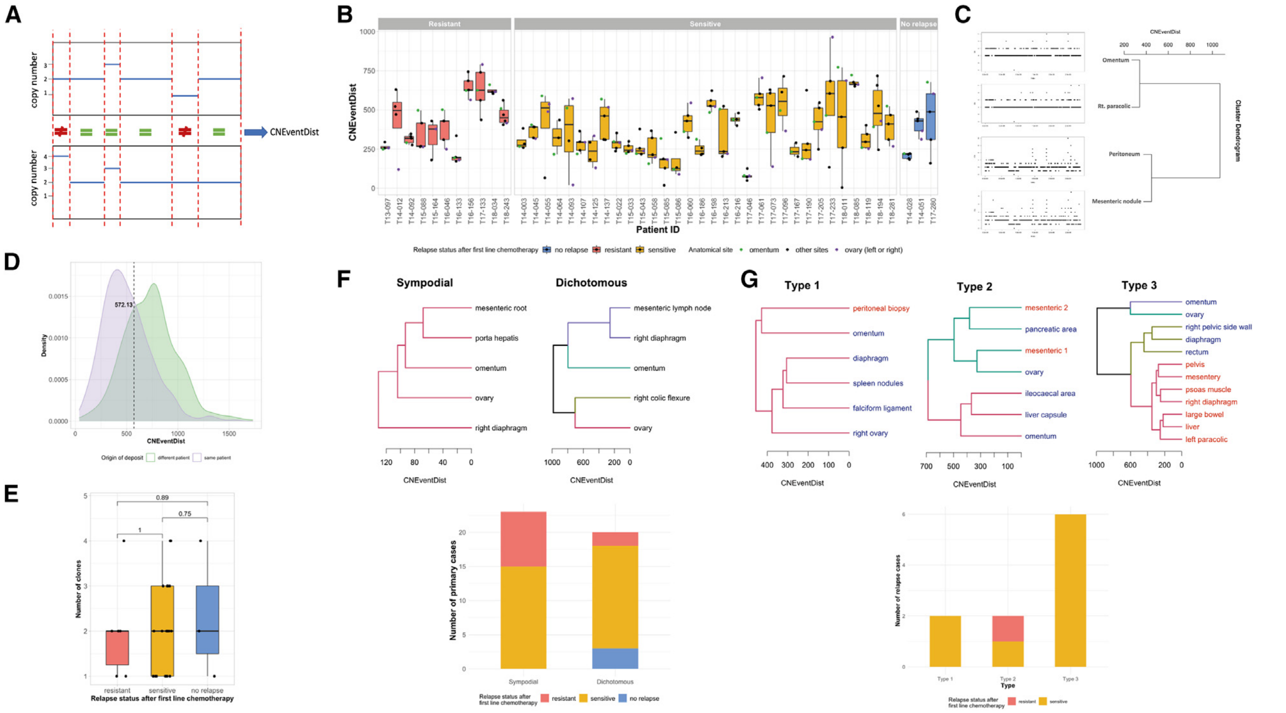

Figure 2. Distinct patterns of genomic heterogeneity and tumor evolution revealed in primary and relapsed HGSOC

(A) CNEventDist derivation. Schematic illustrating the calculation of number of CNEventDists between tumor deposit genomes. Two hypothetical genome-wide total CN profiles are shown, one above the other. Breakpoints in either sample are indicated with the vertical dashed red lines. Each segment between this merged set of breakpoints is classified as either equal (=) or not equal (≠) based on whether the total copy number from the two genomes is the same or not. The CNEventDist is the total number of not-equal segments, in this example, 2.

(B) Genomic heterogeneity of primary tumors. Boxplots showing the number of CNEventDists between tumor samples from various anatomical sites and their matched germline sample across 46 patients, grouped by relapse status.

(C) CNEventDists reveal evolutionary history of a tumor. Genome-wide total copy-number profiles are shown for four deposits from primary debulking of a single patient (vertically stacked on left-hand side). These profiles are matched to the corresponding deposit in the dendrogram resulting from hierarchical clustering of the CNEventDists between the profiles (right-hand side). In this example, the four deposits clearly separate into two clusters of two tumor samples, with similar genomes within a cluster and distinct genomes between clusters.

(D) Defining clonal CNA heterogeneity. Density plot showing the distribution of the number of CNEventDists between tumor deposits from the same patient (intra-patient) and between tumor deposits from different patients (inter-patient). Dashed line indicates the threshold that was determined for calling distinct clones within an individual patient (572.3).

(E) Heterogeneity of primary tumor clonal diversity. The number of clones by response grouping for patients with profiles from five tumor sites profiled (n = 30). Kruskal-Wallis test, p = 0.9454.

(F) Heterogeneous clustering patterns of tumor evolution for primary presentation cases based on inter-deposit CNEventDists. Dendrograms showing representative sympodial (left, case T17-046) and dichotomous (right, case T18-011) clustering patterns from two different primary cases. Each color shown on the dendrogram branches indicates a different clone. Bar chart (lower) showing the prevalence of the two different clustering patterns (topologies), grouped by relapse status; n = 43, because topology could not be determined for three patients. Fisher's exact test, p = 0.03525.

(G) Heterogeneous clustering patterns of tumor evolution with matched relapse samples based on inter-deposit CNEventDists. Representative dendrograms for three different clustering patterns for primary and relapse samples from the same patient. The dendrogram branches are again colored for different clones. Blue text indicates tumor deposits collected from the primary surgery, whereas red text indicates tumor deposits collected at relapse. Type 1 pattern (left) is those with monoclonality in the primary samples, and all relapse samples are from the same clone; type 2 (middle) is polyclonal primary samples and relapse samples originating from one or more of those clones; type 3 (right) shows monoclonality or polyclonality in the primary samples, and at least one relapse sample originates from a clone not present in the primary samples. Bar chart (lower) shows the prevalence of distinct evolutionary relapse patterns.

pattern was observed between samples regardless of which clone was present. Tumors with the same clone had more similar signature profiles than sites with different clones. For example, case T14-137 (Figure 3E) has variable CN signature profiles in the primary tumor samples (blue text), with ovary and omentum clustering together (cluster 1) and showing similar signature profiles (some S6, little to no S3), but differ from the diaphragm, right pelvic side wall, and rectum tumor deposits, which belong to cluster 2. The relapse samples (red text) show similarity to each other in both the CN signature profiles and genomic distances (a single distinct clone that is more similar to cluster 2 than cluster 1). Overlaying the CN signature profiles onto the genomic distances suggests that three distinct clones were present at primary presentation, one of which appears HR repair deficient as indicated by a dominant signature 3, and the patient's relapse is likely to have evolved from that clone.

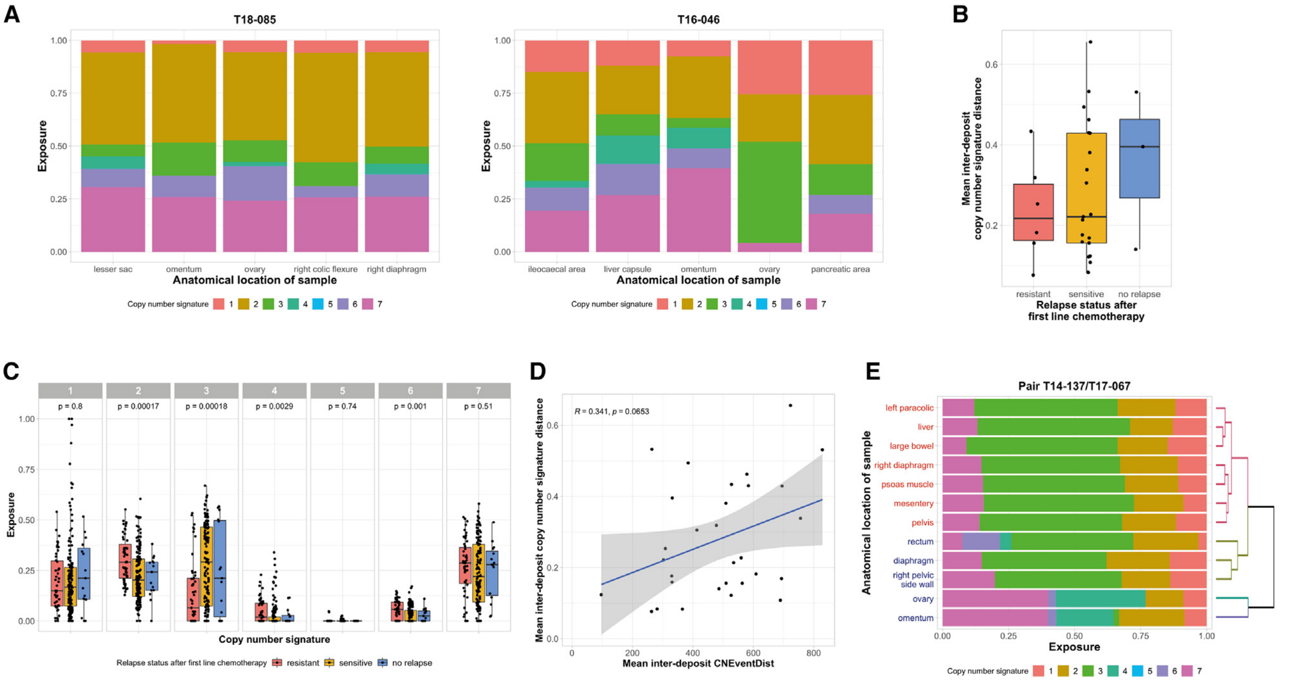

Figure 3. Copy-number signature exposures of disseminated HGSOC demonstrate extensive genomic heterogeneity

(A) Heterogeneous mutational processes that shape tumor evolution. Stacked bar plots showing the copy-number signature exposures for primary tumor samples from two representative sensitive relapse patients. T18-085 (left) has low inter-deposit heterogeneity, meaning there is only a small difference in the copy-number signature exposures when comparing deposits from the same patient, and T16-046 (right) has high inter-deposit heterogeneity.

(B) Variation in copy-number signature heterogeneity. Boxplot showing the mean inter-deposit copy-number signature distance for patients with five tumor samples profiled (n = 30), grouped by the relapse status; Kruskal-Wallis test; p = 0.7084.

(C) Variation in exposure to HGSOC copy-number signatures. Boxplot showing the exposure levels of the seven copy-number signatures of HGSOC for 215 samples from 46 patients, grouped by relapse status. Kruskal-Wallis test was performed across samples from patients of different relapse status within each copy-number signature group. Statistical significance was observed for signatures 2 (related to poor overall survival, due to tandem duplication through CDK12 inactivation; p = 0.00021), 3 (related to favorable overall survival, mechanism of BRCA1/2-related HRD; p = 0.0011), 4 (related to poor overall survival, due to whole-genome duplication related to failure of cell-cycle control and phosphatidylinositol 3-kinase (PI3K) inactivation; p = 0.00047), and 6 (related to poor overall survival, mechanism proposed of focal amplification due to failure of cell-cycle control; p = 0.00032).

(D) Relationship between heterogeneity of genomes and heterogeneity of mutational processes. Scatterplot showing the relationship between the mean inter-deposit CNEventDist and the copy-number signature distance for patients with five samples profiled (n = 30). A positive correlation (r = 0.351, p = 0.0653) is observed, indicating a patient with deposits that are more genomically heterogeneous between each other at the copy-number level are shaped by more heterogeneous mutational processes.

(E) Illustration of tumor evolution under distinct mutational processes. Stacked bar plot showing the CN signature exposures for primary and relapsed tumor samples from a patient who relapsed in a sensitive time frame. Samples are clustered using the CNEventDist, with colored branches of the dendrogram indicating each distinct clone estimated in this patient (T14-137). Blue text indicates tumor deposits collected from the primary surgery, and red text indicates tumor deposits collected from the corresponding relapse surgery. Distinct CN signature profiles can be observed in the primary tumor samples in which the samples from the ovary and omentum are similar and cluster together but are different from the samples from the rectum, diaphragm, and the right pelvic side wall, which form another cluster. Clonal origin estimation reveals the relapse samples evolving from the clone represented by the samples from the rectum, the diaphragm, and the right pelvic side wall.

Importantly, this contribution of *BRCA*-related HRD would not have been detected if only a biopsy from the ovary or omentum had been tested.

Clinically relevant biomarkers in HGSOC exhibit intra-tumor heterogeneity

Quantifying tumor HR deficiency (HRD) scores is now standard of care for ovarian cancer patients to determine their suitability for poly-ADP ribose polymerase (PARP) inhibitor-based treatment. We applied an SNP array-based method to generate a genomic instability score for the HR status[36] of each tumor sample within our cohort to identify whether HR scores are uniform in disseminated HGSOC. HR scores for 199 tumors from 45 patients with aberrant cell fractions >30% were determined (Figure S4A), utilizing a cutoff <42 to define HR proficient (HRP) and ≥42 for HRD based on previous studies.[37,38] All tumors from all patients with known germline *BRCA1/2* mutations (n = 9, denoted with an asterisk) had HR scores >50, and 21 cases without known germline mutations were also considered HRD (Figure 4A). Only four patients presented with all tumors defined as HRP; three had relapsed in a resistant time frame. Importantly, we observed that 10 patients across the cohort had mixed HR status displaying both HRP and HRD scores across their profiled tumors (Figure 4A). Among these, six were regarded as

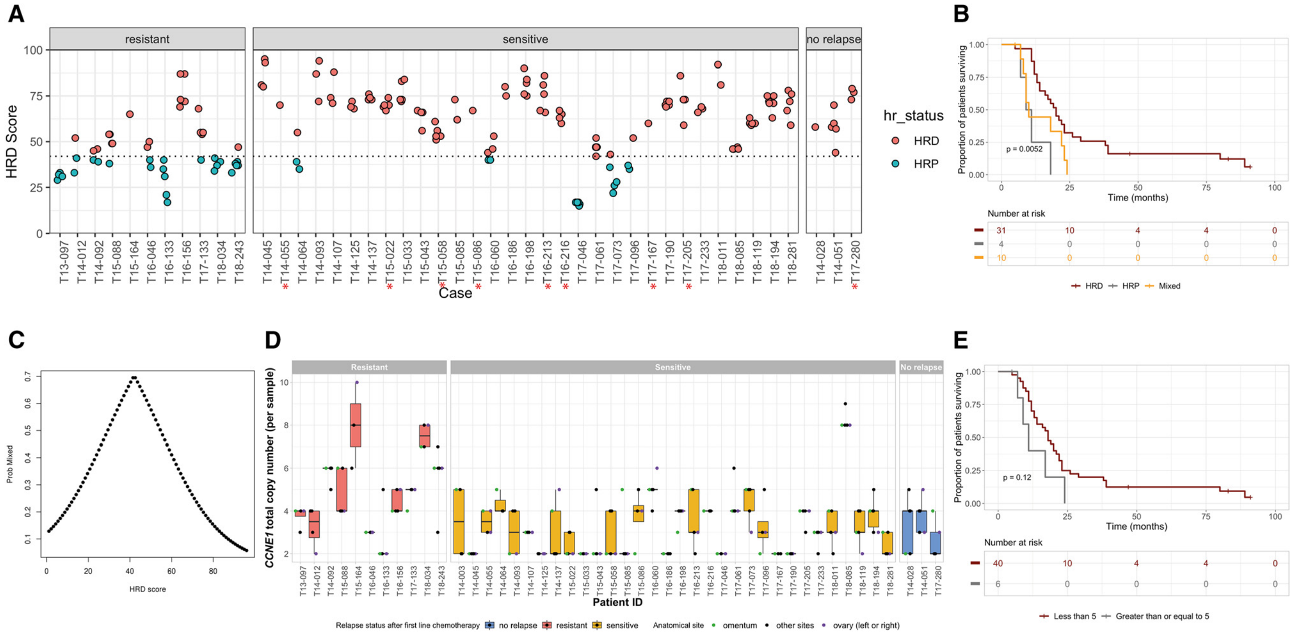

Figure 4. Clinically relevant biomarkers of HGSOC exhibit intra-tumor heterogeneity

(A) Intra-tumor heterogeneity of HR scores. HR scores were calculated for all tumors and plotted as either HRD $\geq$ 42 (red) or HRP < 42 (blue). Patients with known germline *BRCA* mutations are highlighted with red asterisks (n = 9). Ten patients within the resistant relapse group (n = 6) and sensitive relapse group (n = 4) present with mixed HR status.

(B) Clinical implication of HR status. Kaplan-Meier curve showing the association between progression-free survival (p = 0.0052) and the three different HR categories: all tumors within a patient with an HRP profile (HRP), all tumors had an HRD profile (HRD), or if there was a mix of HRP and HRD profiles within a patient's tumors (mixed).

(C) Probability of ambiguous HR designation across multiple deposits. We show predicted probability of contradictory HR status calls (HR score < 42 vs. HR $\geq$ 42) as a function of HR score. Predicted probabilities come from a logistic regression model fitted to the observations across all samples: for each sample, there was another sample from the same patient with HR score on the opposite side of the HRD threshold of 42.

(D) Intra-tumor heterogeneity of *CCNE1* CN. Boxplots showing *CCNE1* total CN per sample across patients (n = 46), grouped by relapse status after first-line chemotherapy, and points colored by anatomical site of each tumor location.

(E) Clinical implication of *CCNE1* amplification. Kaplan-Meier curve showing the association between the mean *CCNE1* total CN across multiple deposits within a patient and progression-free survival, with patients stratified based on the mean *CCNE1* CN of greater or less than five copies (p = 0.12).

treatment resistant and four sensitive. We did not observe a relationship between classification of HRD/HRP and tumor purity, suggesting that contamination of samples with normal cells did not contribute to a mixed pattern of classification within a patient (Figure S4A).

We examined whether a mixed HR status was associated with survival and found that those with a mixed HR status or HRP status had a poorer PFS (p = 0.0052; Figure 4B) and OS (p = 0.00092; Figure S4D) than patients with all HRD tumors (Table S1B). No patients with germline BRCA mutations had tumors of mixed status. Two of the cases with relapse samples had mixed HR status for their primary tumors, and despite the anticipated selective pressure of treatment, the relapse samples from these cases were also of mixed status (Figure S4B). We investigated the mixed HR status in the context of tumor evolution from primary to relapse as determined by our genomic distances in case T17-096 and observed that the tumors clustered by HR status (Figure S4C). In patients of mixed status, HR scores tended to fall within ±10 of the HR score cutoff of 42; therefore, we used a logistic regression model to quantify the relationship between HR score from a single deposit and the chance of the patient containing other deposits with contrasting HR status. This model shows that the probability of an HR measurement obtained from a single deposit not being reproduced in another deposit from the same patient peaks as the HR score approached the threshold of 42, and remarkably, at this value the probability is approximately 70% (Figure 4C).

Amplification of *CCNE1*, encoding the cell-cycle regulator cyclinE1, is associated with poor outcome or development of resistance in HGSOC.[12,39,40] In our cohort, we observed that *CCNE1* CN varies in primary disease across multi-site deposits within a patient and within the cohort (Figure 4D). Applying the COSMIC definition for copy-gain or loss relative to ploidy (https://cancer.sanger.ac.uk/cosmic/help/cnv/overview), we observed *CCNE1* CN amplification in a subset of the multi-site tumors from four patients (Figures S5A and S5B; Table S1F). Survival analysis demonstrated that patients with a mean *CCNE1* CN of five or more copies have a non-significant shorter PFS (p = 0.12; Figure 4E) and OS (p = 0.094; Figure S4E). Differences in *CCNE1* CN were also observed from primary presentation to relapse (Figure S4F). Additional genes where CN levels are known to vary in HGSOC were assessed to determine whether this intra-tumor heterogeneity in CN occurs for other genes in our cohort, for example, *MYC* is frequently amplified in HGSOC

cases.[19,41–43] Similar to *CCNE1*, heterogeneity in *MYC* CN was detected at primary presentation and from primary to relapse (Figures S5A and S5B; Table S1F), but no association with survival outcomes was observed (Figures S4G and S4H). The tumor suppressor genes *PTEN* and Neurofibromin 1 (*NF1*) were also examined, and both demonstrated wide CN variations across tumors (Figures S5A and S5B; Table S1F). Heterogeneity in CN for *MYC*, *PTEN*, and *NF1* was also identified relative to ploidy and aberrant cell fraction (Figures S5A and S5B; Table S1F).

Mutational analysis of disseminated HGSOC

HGSOC is predominantly driven by CN alterations, rather than single-nucleotide variants (SNVs) and INDELs, except for those affecting the *TP53*, *CDK12*, and HR-related genes. However, some SNVs can play important roles in HGSOC biology. For a subset of cases, we analyzed 18 genes (Table S1C) recurrently mutated in HGSOC[18,21] in germline DNA, primary and paired relapse samples (Figures S4A and S4B; Tables S1G and S1H). Other than *TP53*, only seven patients demonstrated different somatic mutations, consistent with previous data (Figure 5A), and there was limited intra-patient heterogeneity in the frequency of SNVs in these genes: three of four patients appeared to have clonal SNVs in *NF1*, *CTNNB1*, or *BARD1*. We highlight one patient, T16-046 (Figure 5B): two samples (ileocecal and pancreatic area) resected at primary surgery featured a *PIK3CA* missense mutation (c.1633G>A), but the omentum, liver capsule, and ovary sites did not. The profiled relapse mesenteric tumor featured the same mutation. As the primary samples clustered into two distinct clones, it was assumed that one of those clones would contain the *PIK3CA*-mutant samples, and the relapse sample would be derived from this clone. However, one *PIK3CA*-mutant sample appeared in each clone of the primary disease. On inspecting the detected frequency of this variant allele in each sample, it was found to be present at low levels in all but the primary ovary tumor. This variant allele was not detected but could have been present at very low levels and positively selected for during the evolution of relapse.

Phenotypic, anatomical, Ki67 proliferation index, and proteomic heterogeneity in disseminated HGSOC tumors

Primary 2D tumor cell cultures were established from disseminated tumors (mean, 9; range, 4–15) for the majority of patients (n = 44 patients, n = 394 tumor cultures) and treated with cisplatin. Phenotypic heterogeneity, measured as induction of apoptosis following treatment with cisplatin, was observed in primary cultures derived from patient tumors. Across the cohort, there was little variation (measured by median absolute deviation [MAD]) observed in apoptotic responses in the different relapse groups (Figure 5C; Figure S6A). Minor variations were observed in responses to platinum treatment across different abdominal areas, with the resistant group showing higher apoptosis scores compared with the sensitive and no-relapse groups in tumors collected from the lower and middle abdomen (Figure S6B). No associations were observed between apoptosis read-outs and genomic distances (Figure S6C). Half maximal inhibitory concentration (IC_{50}) assays were also established for cisplatin drug treatment in a subset of cases (n = 21 patients, n = 154 tumor cultures); however, no significant associations were detected between MAD cisplatin IC_{50} values and relapse status, or cisplatin IC_{50} values and genomic distances (Figure S6D; Tables S1I and S1J).

Extensive intraoperative mapping to IMO grid of disseminated tumors mapped all disseminated tumor samples to an anatomical location.[29] We used Mantel test statistics and observed a tendency for the anatomical distance between tumor deposits to be positively correlated with the derived genomic distances between tumors (STAR Methods). We illustrate such a correlation with case T15-058 ($r = 0.784$, $p = 0.02$; Figure 5D). Tumors formed two clusters on the basis of genomic distance: samples from the splenic lymph node (LN) and right diaphragm located in the upper abdomen clustered together, whereas samples from omentum (middle abdomen), rectal wall, and ovary (lower abdomen) formed another distinct cluster.

Targeted proteomic profiles (297 proteins) were determined in a small pilot subset of patients (n = 7) by reverse phase protein array from primary tumor cultures. We hypothesized that proteins responsible for tumor identity would show greater homogeneity of expression within genomically similar clones and heterogeneity of expression between distinct clones, manifesting in correlations between genomic distances and protein expression differences (Table S1D). A significant positive correlation ($r = 0.33$, $p = 0.0003$) between genomic distance and protein expression change was observed for Cyclin D1, indicating that tumors that share high Cyclin D1 protein expression tend to share similar CN profiles (Table S1D). Further, such statistically significant correlations were observed between expression of other proteins, including GATA3, Src, and BRD4, and genomic heterogeneity (full list of 16 proteins is in Table S1D).

The Ki67 proliferative index of tumors is applied as a prognostic biomarker in breast cancer and melanoma.[44–46] However, the prognostic value of evaluating a Ki67 proliferative index for HGSOC patients is unclear with conflicting reports.[47–51] However, previous attempts assessed only a single tumor site per patient. Scoring for Ki67 was performed on frozen sections adjacent to those used for molecular analyses, with categories defined based on the distribution of the scoring data (<20%, 20%–60%, >60%; Figure S6E). Variations in proliferative index scores were observed, with 32.6% of patients having primary and disseminated tumors scoring within all three categories, and 45.6% of patients with scores in two categories (Figure 5E), suggesting that the assessment of Ki67 as an accurate prognostic biomarker for HGSOC is confounded by extensive variation between tumor sites. In keeping with this, we found no association between Ki67 proliferative index and anatomical location or survival (Figures S6F–S6I).

DISCUSSION

A complex picture of tumor heterogeneity has begun to emerge for HGSOC. Increasing our understanding of the evolution of HGSOC from primary presentation to relapse is paramount, because despite improvements in treatments, overall cure rates of HGSOC have not improved. In this study, we decoded intratumoral heterogeneity in HGSOC at an extensive level, analyzing primary and multiple distant metastases, as well as recurrent

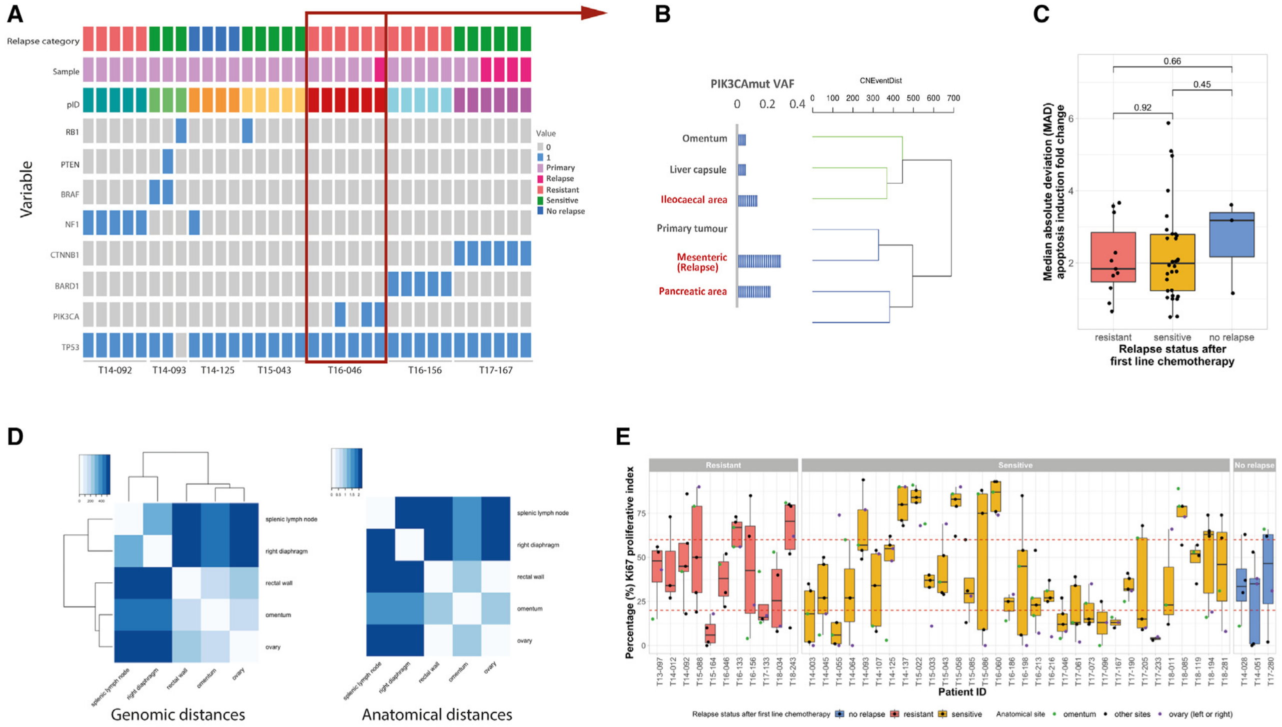

Figure 5. Heterogeneity of disseminated HGSOC displayed by genomic mutational profiling, phenotypic, anatomic, and proteomic analysis

(A) Profile of somatic driver gene mutations in primary and relapse tumors. Oncoprint showing SNVs for selected driver genes (*TP53*, *BRCA1*, *BRCA2*, *CCNE1*, *NF1*, *RB1*, *PTEN*, *BRAF*, *CTNNB1*, *BARD1*, and *PIK3CA*) in each deposit from patients whose tumor samples showed some somatic SNVs affecting these genes. Samples (columns) are ordered by patient and additionally labeled with the corresponding patient's relapse category, and whether the sample was taken at primary debulking surgery or at relapse. Presence of any SNVs is indicated by blue boxes. Most patients showed SNVs that were clearly either clonal (detected in all deposits) or sporadic (only detected in one deposit). Patient T16-046 is highlighted, because the *PIK3CA* SNV was detected in some, but not all, primary deposits but was also detected in the relapse tumor.

(B) Variable detection of a sub-clonal SNV in *PIK3CA*. The dendrogram representing genomic similarity of tumor deposits from patient T16-046 is shown (right), with branches colored by predicted clone of each tumor. Left, the detected variant allele frequency (VAF) for the *PIK3CA* SNV (c.1633G>A) is shown for each corresponding tumor deposit. Tumor sites are given, with the one sequenced relapse sample labeled. Although the *PIK3CA* SNV was called in only three of the tumor sites (those with red text labels), the same SNV was detected below the filtering threshold in three more tumor deposits. No reads supporting this SNV were detected in the primary ovarian tumor. This suggests that perhaps this patient's tumor evolution is even more complex than revealed by the genome-wide copy-number profiling, with the apparent tumor clones in this patient's disease being composed of multiple sub-clones, some of which carry the *PIK3CA* SNV and some of which do not.

(C) Functional heterogeneity of primary tumor cells. Boxplot showing the median absolute deviation (MAD) of sample-wise apoptosis induction fold change of primary cell cultures that were grown from tumor deposits from patients (n = 44; range, 4–15 samples per patient), grouped by relapse status. Kruskal-Wallis test, p = 0.3338.

(D) Relating genomic diversity between tumor deposits to anatomical distance. Heatmap of distance matrix derived from genomic distances (left) and anatomical distances (right) of a representative case (T15-058) showing positive and significant correlation between genomic and anatomical distances of tumor deposits from five anatomical sites (r = 0.784, p = 0.02).

(E) Heterogeneity of proliferation index given by Ki67 scores in disseminated HGSOC tumors. Boxplot showing the percentage Ki67 proliferation index between tumor samples from various anatomical sites across 46 patients, grouped by relapse status. Red dotted lines indicate boundaries for low-, moderate-, and high-percentage Ki67 proliferative index score categories.

tumors through multi-omics and functional assessment. We were able to demonstrate widespread spatial and temporal tumoral heterogeneity at a genomic (CN), functional, anatomical, and proteomic level in patients with HGSOC. The combination of detailed molecular and functional characterization of a large number of HGSOC patients, applied to samples obtained through systematic surgical multi-site collection with lengthy clinical follow-up, represents interesting features of our study.

We identified three pathways of development of relapse, which appear to be consistent with previous studies from other groups. Previous phylogenetic analysis of multiple tumors from a smaller number (n = 7) of HGSOC patients at primary presentation identified distinct modes of clonal spread and intraperitoneal dissemination. Patients displayed either monoclonal and unidirectional seeding or polyclonal spread, indicating the spectrum of clonal migration in HGSOC.[52] In another study, early divergence events were responsible for relapse in two HGSOC patients with high levels of clonal expansion.[18] Within our larger cohort we also observed variations in clonal diversity and migration. By quantifying genomic divergence between tumors, we were able to demonstrate cases of monoclonality versus extensive polyclonality. When examining the phylogeny of tumor

evolution across patients, most resistant patients displayed a simpler sympodial branching pattern, which suggests that a dominant clone expanded relatively early in the tumor's development within the peritoneum of these patients. Within the sensitive relapse group, patients were equally split into either sympodial or dichotomous patterns of tumor evolution. Importantly, three distinct patterns of tumor clonal evolution were observed in patients with matched relapse samples, which implied that in more than half of the patients profiled, at least one of the relapse tumors could be considered clonally distinct from the primary disease. These findings strongly indicate that the evolution of relapsed disease does not appear to be homogeneous and may arise from single, multiple, or newly identified clones within the same patient.

By applying well-defined CN signatures[21] to our cohort, we further explored the extensive genomic complexity across disseminated disease at both presentation and relapse. We demonstrated an enrichment of different signatures related to distinct genomic events and outcomes within the same patient, thus confirming the complexity of HGSOC genomes across the entire disease burden, which would not have been naturally evident through single-tumor profiling. We observed that tumor signature exposure scores related to poor outcome were significantly enriched in those patients who progressed on treatment or relapsed early. In contrast, we detected mainly elevated signature 3 exposure scores within the sensitive relapse and no-relapse groups, indicating a favorable association. Nevertheless, no significant associations were observed between signature exposures and clinical parameters, such as postoperative residual disease or tumor dissemination patterns. A positive correlation was observed between genomic distances and CN signatures. In the tumors that depart from this trend, it was relatively common to find a high genomic heterogeneity arising despite consistent between-sample signature patterns and inferred homogeneous mutational processes. By contrast, heterogeneity in mutational processes nearly always resulted in heterogeneous genomes, implying that it is unlikely that patients with deposits sharing relatively consistent genomes arose from distinct mutational processes (i.e., distinct but convergent evolution). A subset of patients showed genomic heterogeneity was profoundly enriched. For example, in the tumors from T16-046, a *PIK3CA* coding mutation was detected with variable allele frequencies across tumor deposits collected at primary presentation and relapse, with apparent positive selection in relapse. However, it was not detected in the primary ovarian tumor sample. At a theoretical level, unless every single cancer cell is profiled, it is difficult to specify the exact time of emergence of any given clone and its role in carcinogenesis and progression.

Numerous international attempts are being conducted to profile ovarian cancer aiming to develop prognostic scores, similar to the Onco-type DX Recurrence Score in breast cancer that predicts risk of relapse and probability of response to chemotherapy and/or hormonal treatment. Because HRD occurs in half of HGSOC patients,[19] and actionable mutations are not common, genomic instability is emerging as a key therapeutic target for ovarian cancer. The introduction of PARP inhibitors (PARPi) has revolutionized the therapeutic management of HGSOC, and current guidelines recommend *BRCA* mutation status and tumor HRD scores be assessed in all HGSOC patients.[28] However, testing for HR status is currently performed on DNA isolated from one tumor block per patient. The spectrum of genomic heterogeneity observed in our cohort, including mixed CN-based signature exposure scores related to HR within individual patients, suggested that HR status may not be a uniform factor for some HGSOC patients. Applying an SNP array-based algorithm[36] to our cohort, we demonstrated that patients with germline *BRCA1/2* mutations all had HRD scores >42 across all tumor sites profiled. However, for the *BRCA1/2* wild-type patients and those with unknown *BRCA* status, we observed that 22% of patients had a mixed HR status, displaying both HRP and HRD scores across their tested tumors. Interestingly, many of the mixed HR scores were within ±10 of the cutoff of 42. A recent study explored revising the threshold to >33,[53] because patients with HR scores <42 had derived some clinical benefit from receiving niraparib in previous studies,[54] and showed that HRD status derived at >33 remained significantly associated with CA125 response. Our data reinforce such an adjustment to a lower threshold to allow possibly more patients to gain access to PARP inhibitors. In contrast, such a shift would in parallel increase the likelihood of more patients deriving no or limited benefit from PARPi treatment.[53] Validation of these mixed HR profiles is essential, including employing clinically validated tests on multi-site samples to corroborate the profiles we observed, and patients with HR scores close to the threshold of 42 should have further disseminated tumors sampled prior to therapy to confirm HR status. In addition, clinical trials evaluating PARPi treatment modalities and resistance should aim to incorporate multi-site tumor profiling in translational studies to fully elucidate the mechanisms behind treatment responses.

At each level of our genomic, proteomic, and phenotypic analysis, it is clear that a single-site biopsy pre-treatment to guide surgical and systemic management cannot accurately profile a patient's tumor. Our findings highlight that understanding the biology of a given patient's HGSOC disease in order to tailor treatment will require measuring molecular characteristics of as much of the tumor burden as possible. Also, with modern designs of pre-operative window studies, our findings could be applied in the recurrent setting, and tumor mapping could be performed at relapse, which may provide sufficient information about the tumors to guide window studies of targeted agents preoperatively, followed by cytoreductive surgery to improve patient outcome under a more personalized umbrella. Coupled with advances in *ex vivo* patient-derived organoids[55,56] or explant cultures[57] from disseminated tumors, testing of therapies on excised multi-site tumor biopsies could provide a platform to guide personalized treatment.

The quality of surgical debulking has been shown to be strongly dependent on surgical expertise, infrastructure, and overall institutional and team effort and resources.[1,30,58–60] Opponents of radical debulking surgery for patients with high tumor burden have claimed that operability and surgical success are mostly dependent on tumor biology and less on surgical training and expertise. Under that more nihilistic perspective, focus is shifted from the expertise of the treating team toward tumor features that are assumed to be non-modifiable but at this stage are not characterized. It is important to note that despite detailed characterization,

our analysis did not identify a tumor biology-related adverse signature or profile associated with patient outcome that would *a priori* preclude complete or optimal tumor debulking.

In conclusion, we have demonstrated extensive spatial and temporal tumoral heterogeneity in high-tumor-burden, advanced HGSOC patients treated within a maximal effort setting. We show that there are multiple pathways leading to the development of relapse, which cannot be explained by just single resistant clones surviving lines of treatment. Our findings in respect to the variation of HR status and genes such as *CCNE1* and *MYC* give a clear signal that a single biopsy to individualize treatment has the potential to significantly under-represent a patient's unique tumor biology. These findings carry important implications for the gynecological oncological community and provide further insight into ongoing questions of how HGSOC tumor biology influences surgical and clinical outcome.

Limitations of the study

There are limitations to our study. The main ones are that this is a unicentric study, without external validation in additional cohorts and with a limited number of patients to draw many statistically significant conclusions. However, the nature of our study, consisting of multiple surgical samples that are then subsequently extensively processed in the laboratory and paired with equivalent samples at relapse, makes a large-scale study with hundreds of patients almost impossible and associated with highly logistical and technical challenges. Even though we have recruited only 49 patients, this is the largest study of its kind due to the high effort associated with this number. We hope that with external validation in the future in more than one center, we can present a higher number of patients.

STAR★METHODS

Detailed methods are provided in the online version of this paper and include the following:

- KEY RESOURCES TABLE
- RESOURCE AVAILABILITY
  - Lead contact
  - Materials availability
  - Data and code availability
- EXPERIMENTAL MODEL AND SUBJECT DETAILS
  - Patient cohort and treatment setting
  - Primary HGSOC tumor cell cultures
- METHOD DETAILS
  - Copy number analysis
  - Sample-wise genomic distances analysis
  - Determining clonality of tumors
  - Copy number signature analysis
  - HR score estimation
  - Mutational analysis
  - Correlations between genomic and anatomical distances
  - Phenotypic apoptosis assays
  - IC_{50} assays
  - Confirmation of primary tumor culture cell populations via Pax8 immunofluorescent staining
  - Proteomic analysis
  - Ki67 proliferative index staining and assessment
- QUANTIFICATION AND STATISTICAL ANALYSIS
  - Statistical analysis

SUPPLEMENTAL INFORMATION

Supplemental information can be found online at https://doi.org/10.1016/j.xcrm.2023.101055.

ACKNOWLEDGMENTS

Tumor tissues and blood samples were provided by the Imperial College Healthcare NHS Trust Tissue Bank. Other investigators may have received samples from these same tissues. The research was supported by the National Institute for Health Research (NIHR) Biomedical Research Centre (BRC) based at Imperial College Healthcare NHS Trust and Imperial College London. The views expressed are those of the author(s) and not necessarily those of the NHS, the NIHR, or the Department of Health. We thank Dr. Steve McAteer for research support; Nona Rama, Rory Smith, and Cristina Realingo from the Experimental Cancer Medicine Center (Hammersmith Campus, Imperial College) and Dr. Tommy Gorgy for tissue collection support; and Irene Rius-Ruiz and Enanga Eko for laboratory and data support. This study was supported by the Myrovlytis Trust (P.C. and C.F.), Rosetrees Trust (P.C.), Ovarian Cancer Action (P.C., C.F., E.A.S., and D.D.L.B.), Imperial Private Healthcare (K.N. and J.P.), and Imperial Health Charity (Ovarian Fund) (P.C., C.F., and C.M.-A.). E.L.C. was supported by NHMRC and VCA. D.D.L.B. was supported by NHMRC. The IMO diagram of Figure 1 and the graphical abstract were generated using BioRender.

AUTHOR CONTRIBUTIONS

C.F., P.C., E.A.S., E.W.C., and D.D.L.B. conceived and designed this study. K.N., J.J.C., and J.P. collected the clinical annotations. P.C., K.N., J.P., R.W., and C.M.-A. prepared the tumor samples for downstream pipelines and performed functional assays. E.W.C. led the bioinformatics analysis. E.W.C., E.L.C., C.H.K., A.P., and K.T. performed the bioinformatics analysis. S.M. performed Ki67 scoring, and J.L.-K. performed statistical analysis of Ki67 data. P.C., E.W.C., E.L.C., I.A.M., J.K., D.D.L.B., and C.F. contributed to the interpretation of data. P.C., E.W.C., and C.F. wrote the manuscript. All authors contributed and edited the manuscript.

DECLARATION OF INTERESTS

C.F. received honoraria from Ethicon, GSK, Astra Zeneca (AZ)/MSD, Tesaro, Clovis, Sequana, and Roche, outside of the submitted work. E.L.C. received honoraria from AZ outside of the submitted work. E.W.C. and E.A.S. are currently paid employees of GSK and hold honorary lecturer positions at Imperial College London. I.A.M. is on advisory boards and received speaker fees for AZ, Clovis Oncology, GSK/Tesaro, Roche, OncoC4, Epsila Bio, Theolytics, and Duke St Bio, all outside the scope of OCTOPUS, and has institutional grant income from Astra Zeneca. J.K. has received consultancy funding from Astra Zeneca, Clovis, and GSK. D.D.L.B. reports research support grants from Roche-Genentech, AstraZeneca, and BeiGene (paid to institution) and personal consulting fees from Exo Therapeutics, which are outside the submitted work.

INCLUSION AND DIVERSITY

We support inclusive, diverse, and equitable conduct of research.

Received: August 27, 2022
Revised: December 2, 2022
Accepted: April 28, 2023
Published: May 22, 2023

REFERENCES

1. Hall, M., Savvatis, K., Nixon, K., Kyrgiou, M., Hariharan, K., Padwick, M., Owens, O., Cunnea, P., Campbell, J., Farthing, A., et al. (2019). Maximal-effort cytoreductive surgery for ovarian cancer patients with a high tumor burden: variations in practice and impact on outcome. Ann. Surg Oncol. *26*, 2943–2951. https://doi.org/10.1245/s10434-019-07516-3.
2. du Bois, A., Reuss, A., Pujade-Lauraine, E., Harter, P., Ray-Coquard, I., and Pfisterer, J. (2009). Role of surgical outcome as prognostic factor in advanced epithelial ovarian cancer: a combined exploratory analysis of 3 prospectively randomized phase 3 multicenter trials: by the Arbeitsgemeinschaft Gynaekologische Onkologie Studiengruppe Ovarialkarzinom (AGO-OVAR) and the Groupe d'Investigateurs Nationaux Pour les Etudes des Cancers de l'Ovaire (GINECO). Cancer *115*, 1234–1244. https://doi.org/10.1002/cncr.24149.
3. Fotopoulou, C., Concin, N., Planchamp, F., Morice, P., Vergote, I., du Bois, A., and Querleu, D. (2020). Quality indicators for advanced ovarian cancer surgery from the European Society of Gynaecological Oncology (ESGO): 2020 update. Int. J. Gynecol. Cancer *30*, 436–440. https://doi.org/10.1136/ijgc-2020-001248.
4. Moore, K.N., Pothuri, B., Monk, B., and Coleman, R.L. (2020). PARP inhibition as frontline therapy in ovarian cancer. Clin. Adv. Hematol. Oncol. *18*, 550–556.
5. Howells, L., Hulbert-Williams, N.J., and Blagden, S.P. (2019). New challenges in psycho-oncology: using drug development methodology to improve survivorship and supportive care intervention trials. Psycho Oncol. *28*, 1362–1366. https://doi.org/10.1002/pon.5100.
6. Lee, Y., Miron, A., Drapkin, R., Nucci, M.R., Medeiros, F., Saleemuddin, A., Garber, J., Birch, C., Mou, H., Gordon, R.W., et al. (2007). A candidate precursor to serous carcinoma that originates in the distal fallopian tube. J. Pathol. *211*, 26–35. https://doi.org/10.1002/path.2091.
7. Bristow, R.E., Tomacruz, R.S., Armstrong, D.K., Trimble, E.L., and Montz, F.J. (2002). Survival effect of maximal cytoreductive surgery for advanced ovarian carcinoma during the platinum era: a meta-analysis. J. Clin. Oncol. *20*, 1248–1259. https://doi.org/10.1200/JCO.2002.20.5.1248.
8. Cooke, S.L., and Brenton, J.D. (2011). Evolution of platinum resistance in high-grade serous ovarian cancer. Lancet Oncol. *12*, 1169–1174. https://doi.org/10.1016/S1470-2045(11)70123-1.
9. Ahmed, A.A., Etemadmoghadam, D., Temple, J., Lynch, A.G., Riad, M., Sharma, R., Stewart, C., Fereday, S., Caldas, C., Defazio, A., et al. (2010). Driver mutations in TP53 are ubiquitous in high grade serous carcinoma of the ovary. J. Pathol. *221*, 49–56. https://doi.org/10.1002/path.2696.
10. Bowtell, D.D.L. (2010). The genesis and evolution of high-grade serous ovarian cancer. Nat. Rev. Cancer *10*, 803–808. https://doi.org/10.1038/nrc2946.
11. Bowtell, D.D., Böhm, S., Ahmed, A.A., Aspuria, P.-J., Bast, R.C., Jr., Beral, V., Berek, J.S., Birrer, M.J., Blagden, S., Bookman, M.A., et al. (2015). Rethinking ovarian cancer II: reducing mortality from high-grade serous ovarian cancer. Nat. Rev. Cancer *15*, 668–679. https://doi.org/10.1038/nrc4019.
12. Patch, A.M., Christie, E.L., Etemadmoghadam, D., Garsed, D.W., George, J., Fereday, S., Nones, K., Cowin, P., Alsop, K., Bailey, P.J., et al. (2015). Whole-genome characterization of chemoresistant ovarian cancer. Nature *521*, 489–494. https://doi.org/10.1038/nature14410.
13. Bashashati, A., Ha, G., Tone, A., Ding, J., Prentice, L.M., Roth, A., Rosner, J., Shumansky, K., Kalloger, S., Senz, J., et al. (2013). Distinct evolutionary trajectories of primary high-grade serous ovarian cancers revealed through spatial mutational profiling. J. Pathol. *231*, 21–34. https://doi.org/10.1002/path.4230.
14. Eckert, M.A., Pan, S., Hernandez, K.M., Loth, R.M., Andrade, J., Volchenboum, S.L., Faber, P., Montag, A., Lastra, R., Peter, M.E., et al. (2016). Genomics of ovarian cancer progression reveals diverse metastatic trajectories including intraepithelial metastasis to the fallopian tube. Cancer Discov. *6*, 1342–1351. https://doi.org/10.1158/2159-8290.CD-16-0607.
15. Jiménez-Sánchez, A., Cybulska, P., Mager, K.L., Koplev, S., Cast, O., Couturier, D.L., Memon, D., Selenica, P., Nikolovski, I., Mazaheri, Y., et al. (2020). Unraveling tumor-immune heterogeneity in advanced ovarian cancer uncovers immunogenic effect of chemotherapy. Nat. Genet. *52*, 582–593. https://doi.org/10.1038/s41588-020-0630-5.
16. Jiménez-Sánchez, A., Memon, D., Pourpe, S., Veeraraghavan, H., Li, Y., Vargas, H.A., Gill, M.B., Park, K.J., Zivanovic, O., Konner, J., et al. (2017). Heterogeneous tumor-immune microenvironments among differentially growing metastases in an ovarian cancer patient. Cell *170*, 927–938.e20. https://doi.org/10.1016/j.cell.2017.07.025.
17. Masoodi, T., Siraj, S., Siraj, A.K., Azam, S., Qadri, Z., Parvathareddy, S.K., Tulbah, A., Al-Dayel, F., AlHusaini, H., AlOmar, O., et al. (2020). Genetic heterogeneity and evolutionary history of high-grade ovarian carcinoma and matched distant metastases. Br. J. Cancer *122*, 1219–1230. https://doi.org/10.1038/s41416-020-0763-4.
18. Schwarz, R.F., Ng, C.K.Y., Cooke, S.L., Newman, S., Temple, J., Piskorz, A.M., Gale, D., Sayal, K., Murtaza, M., Baldwin, P.J., et al. (2015). Spatial and temporal heterogeneity in high-grade serous ovarian cancer: a phylogenetic analysis. PLoS Med. *12*, e1001789. https://doi.org/10.1371/journal.pmed.1001789.
19. Cancer Genome Atlas Research Network (2011). Integrated genomic analyses of ovarian carcinoma. Nature *474*, 609–615. https://doi.org/10.1038/nature10166.
20. Legendre, P., Fortin, M.-J., and Borcard, D. (2015). Should the Mantel test be used in spatial analysis? Methods Ecol. Evol. *6*, 1239–1247. https://doi.org/10.1111/2041-210X.12425.
21. Macintyre, G., Goranova, T.E., De Silva, D., Ennis, D., Piskorz, A.M., Eldridge, M., Sie, D., Lewsley, L.A., Hanif, A., Wilson, C., et al. (2018). Copy number signatures and mutational processes in ovarian carcinoma. Nat. Genet. *50*, 1262–1270. https://doi.org/10.1038/s41588-018-0179-8.
22. Zhang, A.W., McPherson, A., Milne, K., Kroeger, D.R., Hamilton, P.T., Miranda, A., Funnell, T., Little, N., de Souza, C.P.E., Laan, S., et al. (2018). Interfaces of malignant and immunologic clonal dynamics in ovarian cancer. Cell *173*, 1755–1769.e22. https://doi.org/10.1016/j.cell.2018.03.073.
23. Graf, R.P., Eskander, R., Brueggeman, L., and Stupack, D.G. (2021). Association of copy number variation signature and survival in patients with serous ovarian cancer. JAMA Netw. Open *4*, e2114162. https://doi.org/10.1001/jamanetworkopen.2021.14162.
24. Heitz, F., Kommoss, S., Tourani, R., Grandelis, A., Uppendahl, L., Aliferis, C., Burges, A., Wang, C., Canzler, U., Wang, J., et al. (2020). Dilution of molecular-pathologic gene signatures by medically associated factors might prevent prediction of resection status after debulking surgery in patients with advanced ovarian cancer. Clin. Cancer Res. *26*, 213–219. https://doi.org/10.1158/1078-0432.CCR-19-1741.
25. Lee, S., Zhao, L., Rojas, C., Bateman, N.W., Yao, H., Lara, O.D., Celestino, J., Morgan, M.B., Nguyen, T.V., Conrads, K.A., et al. (2020). Molecular analysis of clinically defined subsets of high-grade serous ovarian cancer. Cell Rep. *31*, 107502. https://doi.org/10.1016/j.celrep.2020.03.066.
26. Millstein, J., Budden, T., Goode, E.L., Anglesio, M.S., Talhouk, A., Intermaggio, M.P., Leong, H.S., Chen, S., Elatre, W., Gilks, B., et al. (2020). Prognostic gene expression signature for high-grade serous ovarian cancer. Ann. Oncol. *31*, 1240–1250. https://doi.org/10.1016/j.annonc.2020.05.019.
27. Colombo, N., and Ledermann, J.A.; ESMO Guidelines Committee Electronic address clinicalguidelines@esmoorg (2021). Updated treatment recommendations for newly diagnosed epithelial ovarian carcinoma from the ESMO Clinical Practice Guidelines. Ann. Oncol. *32*, 1300–1303. https://doi.org/10.1016/j.annonc.2021.07.004.
28. Miller, R.E., Leary, A., Scott, C.L., Serra, V., Lord, C.J., Bowtell, D., Chang, D.K., Garsed, D.W., Jonkers, J., Ledermann, J.A., et al. (2020). ESMO recommendations on predictive biomarker testing for homologous

recombination deficiency and PARP inhibitor benefit in ovarian cancer. Ann. Oncol. *31*, 1606–1622. https://doi.org/10.1016/j.annonc.2020.08.2102.

29. Sehouli, J., Könsgen, D., Mustea, A., Oskay-Ozcelik, G., Katsares, I., Weidemann, H., and Lichtenegger, W. (2003). ["IMO"–intraoperative mapping of ovarian cancer]. Zentralbl. Gynakol. *125*, 129–135. https://doi.org/10.1055/s-2003-41864.

30. Sehouli, J., Senyuva, F., Fotopoulou, C., Neumann, U., Denkert, C., Werner, L., and Gülten, O.O. (2009). Intra-abdominal tumor dissemination pattern and surgical outcome in 214 patients with primary ovarian cancer. J. Surg. Oncol. *99*, 424–427. https://doi.org/10.1002/jso.21288.

31. Querleu, D., Planchamp, F., Chiva, L., Fotopoulou, C., Barton, D., Cibula, D., Aletti, G., Carinelli, S., Creutzberg, C., Davidson, B., et al. (2016). European Society of Gynaecologic Oncology quality indicators for advanced ovarian cancer surgery. Int. J. Gynecol. Cancer *26*, 1354–1363. https://doi.org/10.1097/IGC.0000000000000767.

32. Fotopoulou, C., Richter, R., Braicu, I.E., Schmidt, S.C., Neuhaus, P., Lichtenegger, W., and Sehouli, J. (2011). Clinical outcome of tertiary surgical cytoreduction in patients with recurrent epithelial ovarian cancer. Ann. Surg Oncol. *18*, 49–57. https://doi.org/10.1245/s10434-010-1245-3.

33. Ciriello, G., Miller, M.L., Aksoy, B.A., Senbabaoglu, Y., Schultz, N., and Sander, C. (2013). Emerging landscape of oncogenic signatures across human cancers. Nat. Genet. *45*, 1127–1133. https://doi.org/10.1038/ng.2762.

34. Hoadley, K.A., Yau, C., Hinoue, T., Wolf, D.M., Lazar, A.J., Drill, E., Shen, R., Taylor, A.M., Cherniack, A.D., Thorsson, V., et al. (2018). Cell-of-Origin patterns dominate the molecular classification of 10,000 tumors from 33 types of cancer. Cell *173*, 291–304.e6. https://doi.org/10.1016/j.cell.2018.03.022.

35. Zeira, R., and Raphael, B.J. (2020). Copy number evolution with weighted aberrations in cancer. Bioinformatics *36*, i344–i352. https://doi.org/10.1093/bioinformatics/btaa470.

36. Sztupinszki, Z., Diossy, M., Krzystanek, M., Reiniger, L., Csabai, I., Favero, F., Birkbak, N.J., Eklund, A.C., Syed, A., and Szallasi, Z. (2018). Migrating the SNP array-based homologous recombination deficiency measures to next generation sequencing data of breast cancer. npj Breast Cancer *4*, 16. https://doi.org/10.1038/s41523-018-0066-6.

37. Telli, M.L., Timms, K.M., Reid, J., Hennessy, B., Mills, G.B., Jensen, K.C., Szallasi, Z., Barry, W.T., Winer, E.P., Tung, N.M., et al. (2016). Homologous recombination deficiency (HRD) score predicts response to platinum-containing neoadjuvant chemotherapy in patients with triple-negative breast cancer. Clin. Cancer Res. *22*, 3764–3773. https://doi.org/10.1158/1078-0432.CCR-15-2477.

38. Timms, K.M., Abkevich, V., Hughes, E., Neff, C., Reid, J., Morris, B., Kalva, S., Potter, J., Tran, T.V., Chen, J., et al. (2014). Association of BRCA1/2 defects with genomic scores predictive of DNA damage repair deficiency among breast cancer subtypes. Breast Cancer Res. *16*, 475. https://doi.org/10.1186/s13058-014-0475-x.

39. Etemadmoghadam, D., deFazio, A., Beroukhim, R., Mermel, C., George, J., Getz, G., Tothill, R., Okamoto, A., Raeder, M.B., Harnett, P., et al. (2009). Integrated genome-wide DNA copy number and expression analysis identifies distinct mechanisms of primary chemoresistance in ovarian carcinomas. Clin. Cancer Res. *15*, 1417–1427. https://doi.org/10.1158/1078-0432.CCR-08-1564.

40. Noske, A., Henricksen, L.A., LaFleur, B., Zimmermann, A.K., Tubbs, A., Singh, S., Storz, M., Fink, D., and Moch, H. (2015). Characterization of the 19q12 amplification including CCNE1 and URI in different epithelial ovarian cancer subtypes. Exp. Mol. Pathol. *98*, 47–54. https://doi.org/10.1016/j.yexmp.2014.12.004.

41. Darcy, K.M., Brady, W.E., Blancato, J.K., Dickson, R.B., Hoskins, W.J., McGuire, W.P., and Birrer, M.J. (2009). Prognostic relevance of c-MYC gene amplification and polysomy for chromosome 8 in suboptimally-resected, advanced stage epithelial ovarian cancers: a Gynecologic Oncology Group study. Gynecol. Oncol. *114*, 472–479. https://doi.org/10.1016/j.ygyno.2009.05.012.

42. Helland, Å., Anglesio, M.S., George, J., Cowin, P.A., Johnstone, C.N., House, C.M., Sheppard, K.E., Etemadmoghadam, D., Melnyk, N., Rustgi, A.K., et al. (2011). Deregulation of MYCN, LIN28B and LET7 in a molecular subtype of aggressive high-grade serous ovarian cancers. PLoS One *6*, e18064. https://doi.org/10.1371/journal.pone.0018064.

43. Wu, R., Lin, L., Beer, D.G., Ellenson, L.H., Lamb, B.J., Rouillard, J.M., Kuick, R., Hanash, S., Schwartz, D.R., Fearon, E.R., and Cho, K.R. (2003). Amplification and overexpression of the L-MYC proto-oncogene in ovarian carcinomas. Am. J. Pathol. *162*, 1603–1610. https://doi.org/10.1016/S0002-9440(10)64294-0.

44. Gimotty, P.A., Van Belle, P., Elder, D.E., Murry, T., Montone, K.T., Xu, X., Hotz, S., Raines, S., Ming, M.E., Wahl, P., and Guerry, D. (2005). Biologic and prognostic significance of dermal Ki67 expression, mitoses, and tumorigenicity in thin invasive cutaneous melanoma. J. Clin. Oncol. *23*, 8048–8056. https://doi.org/10.1200/JCO.2005.02.0735.

45. Torres-Cabala, C., Li-Ning-Tapia, E., and Hwu, W.J. (2020). Pathology-based biomarkers useful for clinical decisions in melanoma. Arch. Med. Res. *51*, 827–838. https://doi.org/10.1016/j.arcmed.2020.09.008.

46. Yerushalmi, R., Woods, R., Ravdin, P.M., Hayes, M.M., and Gelmon, K.A. (2010). Ki67 in breast cancer: prognostic and predictive potential. Lancet Oncol. *11*, 174–183. https://doi.org/10.1016/S1470-2045(09)70262-1.

47. Bachmayr-Heyda, A., Aust, S., Heinze, G., Polterauer, S., Grimm, C., Braicu, E.I., Sehouli, J., Lambrechts, S., Vergote, I., Mahner, S., et al. (2013). Prognostic impact of tumor infiltrating CD8+ T cells in association with cell proliferation in ovarian cancer patients–a study of the OVCAD consortium. BMC Cancer *13*, 422. https://doi.org/10.1186/1471-2407-13-422.

48. Chen, M., Yao, S., Cao, Q., Xia, M., Liu, J., and He, M. (2017). The prognostic value of Ki67 in ovarian high-grade serous carcinoma: an 11-year cohort study of Chinese patients. Oncotarget *8*, 107877–107885. https://doi.org/10.18632/oncotarget.14112.

49. Darb-Esfahani, S., Kolaschinski, I., Trillsch, F., Mahner, S., Concin, N., Vergote, I., Van Nieuwenhuysen, E., Achimas-Cadariu, P., Glajzer, J., Woopen, H., et al. (2018). Morphology and tumour-infiltrating lymphocytes in high-stage, high-grade serous ovarian carcinoma correlated with long-term survival. Histopathology *73*, 1002–1012. https://doi.org/10.1111/his.13711.

50. Khouja, M.H., Baekelandt, M., Nesland, J.M., and Holm, R. (2007). The clinical importance of Ki-67, p16, p14, and p57 expression in patients with advanced ovarian carcinoma. Int. J. Gynecol. Pathol. *26*, 418–425. https://doi.org/10.1097/pgp.0b013e31804216a0.

51. Qiu, D., Cai, W., Zhang, Z., Li, H., and Zhou, D. (2019). High Ki-67 expression is significantly associated with poor prognosis of ovarian cancer patients: evidence from a meta-analysis. Arch. Gynecol. Obstet. *299*, 1415–1427. https://doi.org/10.1007/s00404-019-05082-3.

52. McPherson, A., Roth, A., Laks, E., Masud, T., Bashashati, A., Zhang, A.W., Ha, G., Biele, J., Yap, D., Wan, A., et al. (2016). Divergent modes of clonal spread and intraperitoneal mixing in high-grade serous ovarian cancer. Nat. Genet. *48*, 758–767. https://doi.org/10.1038/ng.3573.

53. Stronach, E.A., Paul, J., Timms, K.M., Hughes, E., Brown, K., Neff, C., Perry, M., Gutin, A., El-Bahrawy, M., Steel, J.H., et al. (2018). Biomarker assessment of HR deficiency, tumor BRCA1/2 mutations, and CCNE1 copy number in ovarian cancer: associations with clinical outcome following platinum monotherapy. Mol. Cancer Res. *16*, 1103–1111. https://doi.org/10.1158/1541-7786.MCR-18-0034.

54. Mirza, M.R., Monk, B.J., Herrstedt, J., Oza, A.M., Mahner, S., Redondo, A., Fabbro, M., Ledermann, J.A., Lorusso, D., Vergote, I., et al. (2016). Niraparib maintenance therapy in platinum-sensitive, recurrent ovarian cancer. N. Engl. J. Med. *375*, 2154–2164. https://doi.org/10.1056/NEJMoa1611310.

55. Hoffmann, K., Berger, H., Kulbe, H., Thillainadarasan, S., Mollenkopf, H.J., Zemojtel, T., Taube, E., Darb-Esfahani, S., Mangler, M., Sehouli, J., et al.

(2020). Stable expansion of high-grade serous ovarian cancer organoids requires a low-Wnt environment. EMBO J. *39*, e104013. https://doi.org/10.15252/embj.2019104013.

56. Kopper, O., de Witte, C.J., Lõhmussaar, K., Valle-Inclan, J.E., Hami, N., Kester, L., Balgobind, A.V., Korving, J., Proost, N., Begthel, H., et al. (2019). An organoid platform for ovarian cancer captures intra- and inter-patient heterogeneity. Nat. Med. *25*, 838–849. https://doi.org/10.1038/s41591-019-0422-6.

57. Meijer, T.G., Naipal, K.A., Jager, A., and van Gent, D.C. (2017). Ex vivo tumor culture systems for functional drug testing and therapy response prediction. Future Sci. OA *3*, FSO190. https://doi.org/10.4155/fsoa-2017-0003.

58. Aletti, G.D., Dowdy, S.C., Gostout, B.S., Jones, M.B., Stanhope, R.C., Wilson, T.O., Podratz, K.C., and Cliby, W.A. (2009). Quality improvement in the surgical approach to advanced ovarian cancer: the Mayo Clinic experience. J. Am. Coll. Surg. *208*, 614–620. https://doi.org/10.1016/j.jamcollsurg.2009.01.006.

59. Aletti, G.D., Eisenhauer, E.L., Santillan, A., Axtell, A., Aletti, G., Holschneider, C., Chi, D.S., Bristow, R.E., and Cliby, W.A. (2011). Identification of patient groups at highest risk from traditional approach to ovarian cancer treatment. Gynecol. Oncol. *120*, 23–28. https://doi.org/10.1016/j.ygyno.2010.09.010.

60. Tseng, J.H., Cowan, R.A., Zhou, Q., Iasonos, A., Byrne, M., Polcino, T., Polen-De, C., Gardner, G.J., Sonoda, Y., Zivanovic, O., et al. (2018). Continuous improvement in primary Debulking surgery for advanced ovarian cancer: do increased complete gross resection rates independently lead to increased progression-free and overall survival? Gynecol. Oncol. *151*, 24–31. https://doi.org/10.1016/j.ygyno.2018.08.014.

61. Van Loo, P., Nordgard, S.H., Lingjærde, O.C., Russnes, H.G., Rye, I.H., Sun, W., Weigman, V.J., Marynen, P., Zetterberg, A., Naume, B., et al. (2010). Allele-specific copy number analysis of tumors. Proc. Natl. Acad. Sci. USA *107*, 16910–16915. https://doi.org/10.1073/pnas.1009843107.

62. Durinck, S., Spellman, P., Birney, E., and Huber, W. (2009). Mapping identifiers for the integration of genomic datasets with the R/Bioconductor package biomaRt. Nature Protocols *4*, 1184–1191.

63. Durinck, S., Moreau, Y., Kasprzyk, A., Davis, S., De Moor, B., Brazma, A., and Huber, W. (2005). BioMart and Bioconductor: a powerful link between biological databases and microarray data analysis. Bioinformatics *21*, 3439–3440.

64. Lawrence M., Huber W., Pagès H., Aboyoun P., Carlson M., Gentleman R., Morgan M., Carey V. (2013). "Software for Computing and Annotating Genomic Ranges." PLoS Computational Biology, 9. https://doi.org/10.1371/journal.pcbi.1003118.

65. Prat, J.; FIGO Committee on Gynecologic Oncology (2014). Staging classification for cancer of the ovary, fallopian tube, and peritoneum. Int. J. Gynaecol. Obstet. *124*, 1–5. https://doi.org/10.1016/j.ijgo.2013.10.001.

66. Fotopoulou, C., Hall, M., Cruickshank, D., Gabra, H., Ganesan, R., Hughes, C., Kehoe, S., Ledermann, J., Morrison, J., Naik, R., et al. (2017). British Gynaecological Cancer Society (BGCS) epithelial ovarian/fallopian tube/primary peritoneal cancer guidelines: recommendations for practice. Eur. J. Obstet. Gynecol. Reprod. Biol. *213*, 123–139. https://doi.org/10.1016/j.ejogrb.2017.04.016.

67. Harter, P., du Bois, A., Hahmann, M., Hasenburg, A., Burges, A., Loibl, S., Gropp, M., Huober, J., Fink, D., Schröder, W., et al. (2006). Surgery in recurrent ovarian cancer: the Arbeitsgemeinschaft Gynaekologische Onkologie (AGO) DESKTOP OVAR trial. Ann. Surg Oncol. *13*, 1702–1710. https://doi.org/10.1245/s10434-006-9058-0.

68. Harter, P., Sehouli, J., Reuss, A., Hasenburg, A., Scambia, G., Cibula, D., Mahner, S., Vergote, I., Reinthaller, A., Burges, A., et al. (2011). Prospective validation study of a predictive score for operability of recurrent ovarian cancer: the Multicenter Intergroup Study DESKTOP II. A project of the AGO Kommission OVAR, AGO Study Group, NOGGO, AGO-Austria, and MITO. Int. J. Gynecol. Cancer *21*, 289–295. https://doi.org/10.1097/IGC.0b013e31820aaafd.

69. Cheng, J., Vanneste, E., Konings, P., Voet, T., Vermeesch, J.R., and Moreau, Y. (2011). Single-cell copy number variation detection. Genome Biol. *12*, R80. https://doi.org/10.1186/gb-2011-12-8-r80.

STAR★METHODS

KEY RESOURCES TABLE

REAGENT or RESOURCE	SOURCE	IDENTIFIER
**Antibodies**		
Rabbit Monoclonal Ki67	Abcam	Cat #ab16667; RRID:AB_302459
Rabbit Monoclonal Pax-8	Abcam	Cat # ab189249; RRID:AB_2801268
Alexa Fluor 488 Goat anti-rabbit	ThermoFisher Scientific	Cat # A11034; RRID:AB_2576217
**Biological samples**		
Disseminated high grade serous ovarian cancer tumors (n = 49 patients)	Imperial College Healthcare NHS Tissue Bank	https://www.imperial.ac.uk/imperial-college-healthcare-tissue-bank/
Blood samples for germline DNA	Imperial College Healthcare NHS Tissue Bank	https://www.imperial.ac.uk/imperial-college-healthcare-tissue-bank/
**Chemicals, peptides, and recombinant proteins**		
ProLong® Gold Antifade Mountant with DAPI	ThermoFisher Scientific	Cat #P36935
Red Blood Cell lysis buffer	Milteny Biotec	Cat #130-094-183
Cisplatin 1 mg/mL sterile concentrate	Hammersmith Hospital Pharmacy (Onco-Tain)	N/A
MTT reagent (3-(4,5-Dimethylthiazol-2-yl)-2, 5-diphenyltetrazolium bromide)	Merck	Cat #M5655
Dispase II (neutral protease, grade II)	Roche (Sigma)	Cat #04942078001
**Critical commercial assays**		
ApoTox-Glo Triplex Assay	Promega	Cat #G6321
**Deposited data**		
Infinium OmniExpress-24 v1.3 BeadChip (SNP genotyping) array somatic data	This paper	EGAS00001007164
**Software and algorithms**		
R version 4.0.1	The R Foundation for Statistical Computing	N/A
R version 4.2.1	The R Foundation for Statistical Computing	N/A
"Allele-Specific Copy number Analysis of Tumors" (ASCAT) package for R	Van Loo et al.[61]	https://github.com/VanLoo-lab/ascat
"biomaRt" Bioconductor package for R	Durinck et al.[62,63]	https://bioconductor.org/packages/release/bioc/html/biomaRt.html
"GenomicRanges" package for R	Lawrence et al.[64]	https://bioconductor.org/packages/release/bioc/html/GenomicRanges.html
Various R packages for data analysis and visualisation		N/A
Identification of copy number signatures	Macintyre et al.[21]	https://bitbucket.org/britroc/cnsignatures/src/master/
"scarHRD" package for R	Sztupinszki et al.[36]	https://github.com/sztup/scarHRD

RESOURCE AVAILABILITY

Lead contact

Requests for further information or data access should be directed to and fulfilled by the lead contact, Dr Paula Cunnea (p.cunnea@imperial.ac.uk).

Materials availability

This study did not generate any new unique reagents.

Data and code availability

Genomic data has been deposited in European Genome-Phenome Archive (EGA) repository, which is hosted by the EBI and the CRG, under accession number EGAS00001007164. Due to the sensitive nature of our patient datasets as regulated by Imperial College Faculty of Medicine guidance on the sharing and publishing of human genetic data, deposited genomic data must be under controlled access. Access to data can be gained for academic use through our Data Access Committee and made available upon request following contact with lead contact (Dr Paula Cunnea). Code generated in R used in this study is available via the HGSOC_ITH repository (https://github.com/edcurry/HGSOC_ITH). Any additional information required to reanalyze the data reported in this work paper is available from the lead contact upon request.

EXPERIMENTAL MODEL AND SUBJECT DETAILS

Patient cohort and treatment setting

All female patients who underwent primary maximal effort debulking surgery due to advanced FIGO stage III or IV[65] HGSOC within the West London Gynecology Cancer Center of Hammersmith Hospital, Imperial College NHS Trust between September 2013 and November 2018 were eligible for this study. Patients who had previous neoadjuvant chemotherapy, concomitant secondary cancers, or non-epithelial histology were excluded. We selected only chemo-naïve patients for this study as previous studies including tumors collected following neo-adjuvant chemotherapy have indicated treatment may introduce a selective bias.[18,25] The project was performed under the Hammersmith and Queen Charlotte's and Chelsea Research Ethics Committee approval and human samples for this research project were banked by the Imperial College Healthcare Tissue Bank (ICHTB). ICHTB is supported by the National Institute for Health Research (NIHR) Biomedical Research Center based at Imperial College Healthcare NHS Trust and Imperial College London. ICHTB is approved by Wales REC3 to release human material for research (22/WA/2836), and the samples for this project (R14142 plus amendments) were issued from sub-collection reference number GYN_HG_13_020, following full patient consent. The procedures involved human participants were done in accordance with the ethical standards of the institutional and/or national research committee and with the principles of the 1964 Declaration of Helsinki and its later amendments or comparable ethical standards. The West London Gynecology Cancer Center in Hammersmith Hospital is a center of excellence for ovarian cancer surgery as certified by the European Society of Gynecological Oncology (ESGO),[3,31] which ensured that all included patients were operated and treated within a maximal effort and highly specialised setting to exclude any bias of suboptimal surgical and overall treatment quality. The surgery for all patients was indicated and approved within a well-established and regulated Multidisciplinary Tumour-board Meeting (MDT), mandatory for every cancer patient treated within the National Health Service (NHS). Indications for upfront debulking were based on ESGO defined criteria for inoperability and surgical selection to identify surgical candidates for upfront surgery who were expected to be debulked to no or minimal residual disease with a reasonable morbidity profile.[31] All surgeries were performed by a specialised and dedicated multidisciplinary team of experts per midline laparotomy and included a hysterectomy with bilateral salpingoophorectomy (where eligible) and infragastric omentectomy, as well as removal of all visible peritoneal and lymphatic disease with all potentially necessary additional procedures such as bowel resections, splenectomy, diaphragmatectomy, liver capsule resection, lesser sac resections, pleurectomy, paracardiac and celiac trunk LN removal. Intraoperative tumor dissemination patterns and tumor burden were systematically documented in each surgical patient, using the well-established IMO (Intraoperative Mapping of Ovarian Cancer) system, developed and validated to obtain an objective and reproducible documentation of ovarian cancer spread (Figure S1E).[29] All patients had a high tumor burden, allowing for collection of sufficient biopsies from multiple sites. Tumors were regularly sampled not only from the primary ovarian mass and omentum, but from the entire peritoneal and extraperitoneal cavity such as the upper abdomen (spleen, lesser sac, celiac trunk, diaphragm/Morison's pouch, liver capsule, retroperitoneal pelvic and paraortic lymph nodes), the bowel, mesentery, parietal and visceral peritoneum, pleura and paracardiac lymph nodes - where affected (Table S1A). The same plan was used for biopsy mapping of all anatomical sites during surgery and buffy coats from blood samples or normal tissue were collected as germline controls.

Postoperative systemic treatment was applied as per the UK guidelines with combination regimen carboplatin and paclitaxel. Patients in the cohort received standard-of-care platinum-based chemotherapy carboplatin (17.4% carboplatin alone), carboplatin in combination with paclitaxel (45.6%) or paclitaxel/bevacizumab (34.7%) for an average of 6 cycles. Carboplatin Mono was administered in case of patients wish or contraindications to paclitaxel. All stage IV and non-tumour-free operated patients were eligible to receive bevacizumab in the absence of other contraindications such as fistulas or cardioembolic events as per licencing by NICE. None of the patients received PARP inhibitors at first line, since they were not approved at the time of the study.

Patients were followed up 3- and then 6-monthly after the first 2 years, for 5 years, as per national guidelines.[66] Clinical history, examination and CA-125 (if the pre-operative value was elevated) were assessed. A CT/MRI-scan was ordered if the above examinations revealed any pathology. Isolated CA-125 elevation was not regarded as a recurrence. Indications for surgery at relapse were 2--fold; either with the aim of cytoreduction as per the DESKTOP criteria in a platinum sensitive time frame[67,68] or with palliative intent due to symptoms such as bowel obstruction that failed conservative treatment. Patients who had a biopsy or surgery at relapse underwent the same systematic mapping of their tumor burden and had multi-site tumor deposits collected as at primary debulking, where available.

The 49 patients were classified into different response groups according to their time of progression from end of first line chemotherapy to date of first relapse. Three patients died in the first 60 days for causes not related to disease progression and were

therefore excluded from further analysis. The refractory group were patients who progressed while on chemotherapy; the resistant group refers to patients who relapsed within 6 months of completion of chemotherapy; patients who relapsed greater than 6 months following completion of chemotherapy were classified as sensitive; and the no relapse group refers to patients who have not relapsed during the follow up period of the study (median 74.7 months, IQR 53.6–86.4). For analysis purposes, the refractory and resistant patients were grouped together as ‘‘resistant’’. Progression-free survival (PFS) was determined as the time interval between the event of interest (cytoreductive surgery) until the first defined event of relapse for a patient. The mean age of patients in the cohort was 62 years (range 32–91; Table 1).

Primary HGSOC tumor cell cultures

Tumor biopsies from multiple sites per patient were collected directly into RPMI 1640 media (Sigma-Aldrich, UK) supplemented with 50 U/mL penicillin and 50 μg/mL streptomycin (Life Technologies, UK) and transported to the laboratory within 15 min where they were dissected in 1 mm pieces and incubated with 2.4 U/mL Dispase II (Roche, UK) for 1h at 37°C, 5% CO_2 shaking gently every 2 min. RPMI media supplemented with 20% (v/v) Fetal Calf Serum (First Link, UK), 50 U/mL penicillin and 50 μg/mL streptomycin, 2 mM L-glutamine (Life Technologies, UK), 2 mM Sodium Pyruvate (Sigma-Aldrich) and 2.5 μg/mL Insulin (Sigma-Aldrich) was added to stop the protease activity. Dissociated tumor suspensions were filtered through a 70 μM filter, followed by centrifuging at 450 × g for 15 min. In order to remove any contaminating erythrocytes, Red Blood Cell lysis Buffer (Milteny Biotec, UK) was used depending on sample tissue size according to manufacturer's instructions. Finally, cells were incubated for 30 min in appropriately sized tissue culture flasks to remove populations of stromal cells by more rapid adherence to plastic, and non-adherent tumor cell populations were transferred to fresh tissue culture flasks and incubated at 37°C, 5% CO_2. Purity of tumor cell cultures was estimated by cell morphology and staining for Pax8-positive tumor cells.

METHOD DETAILS

Copy number analysis

Single nucleotide polymorphism (SNP) genotyping was performed on 305 samples from 49 primary including the 10 paired relapse cases using the Infinium OmniExpress-24 v1.3 BeadChip array (Australian Genome Research Facility, Melbourne, Australia) to identify copy number aberrations (CNAs). For each case, a maximum of 5 tumor samples collected at primary presentation, any tumors collected at relapse, and a buffy coat or normal tissue sample were genotyped. The annotation file for each probe was downloaded from the Illumina website (https://www.illumina.com/). For all samples, the fraction of aberrant cells and the tumor ploidy were estimated, and the allele-specific copy number of both parental alleles for 714,238 SNPs across the whole genome was subsequently calculated using the ASCAT algorithm,[61] implemented in the "ASCAT" package in R. Wave correction was performed based on the GC content of Illumina OmniExpress arrays,[69] and germline genotypes were predicted from the HumanOmniExpress-12 platform. 18 tumor samples from 16 cases that were classified as non-aberrant samples, and 1 sample where ASCAT could not determine an optimal ploidy and cellularity value, were removed from further analysis. The sum of the copy number of the major and minor alleles for each SNP were extracted. All SNPs were mapped to the human genome assembly GRCh37 in Ensembl using the "biomaRt" package in R, and a total of 30,972 genes were annotated. As copy numbers of each gene were detected with multiple probes, the mean copy number was taken to represent the allele-specific CN for each individual gene.

Sample-wise genomic distances analysis

To compute the genomic distance between any pair of samples, segmented CN profiles output by ASCAT were compared as summarized in Figure 2A. All breakpoint co-ordinates in either sample were collated, and total CN values for each sample were mapped onto the merged set of segments. The sum of differences between the samples across all segments then represents the estimated number of events required to separate the two samples from a common ancestor. We refer to this measure as the 'Copy Number Event' distance, or 'genomic distance' between any pair of samples. A software implementation of this measure is provided in the HGSOC_ITH repository (https://github.com/edcurry/HGSOC_ITH).

The median intra-patient number-of-CNA-event distance is 469 (range = 68–1,550), whereas the median inter-patient number-of-CNA-event distance is 734.5 (range = 38–1,731). Patterns of tumor evolution for each patient were generated with agglomerative hierarchical clustering with complete linkage, using the CNEvent distances between each pair of samples.

Determining clonality of tumors

Collections of genomic distances were computed to reflect pairs of samples obtained from the same patient (intra-patient) and pairs of samples obtained from different patients (inter-patient). A logistic regression model was fitted to this data and used to estimate the probability of a pair of samples being obtained from different patients, based on the genomic distance between the samples. From this model, we identified the minimum genomic distance above which the predicted probability of a pair of samples deriving from different patients was greater than the predicted probability of the pair of samples deriving from the same patient. This threshold was applied to each patient's tumor deposits, calling sets of deposits distinct clones if they were as divergent as would be expected for deposits from different patients.

Copy number signature analysis

Seven signatures of mutational processes reflected in CN profiles were recently reported for HGSOC.[21] To quantify the copy number signature exposures in the current cohort, the published code from (https://bitbucket.org/britroc/cnsignatures)[21] was applied to ASCAT output for all genotyped samples obtained from our patient cohort. This provides 7 values for each sample, a relative contribution of each signature to the sample's CN aberrations (Table S1E). To summarize the intra-tumoural heterogeneity of CN signatures for each patient, we computed the average Euclidean (sum-squared) distance between each pair of samples from that patient.

HR score estimation

HR deficiency was estimated using the scarHRD R package.[36] Patients who died not due to disease progression were excluded. Tumor samples with ASCAT aberrant cell fraction of <0.3 were excluded, as were those samples with an ASCAT aberrant cell fraction of 1, and values of 0 for HRD-LOH, LST and telomeric AI. Tumors were classified as HR deficient if the sum of the three genomic scar scores, i.e. the HRD Sum score, was ≥ 42.

Mutational analysis

Germline and tumor DNA, including corresponding relapse tumors, from n = 21 patients were profiled. Sequencing libraries were prepared using the Illumina TruSeq DNA Nano Kit at the Australian Genome Research Facility (AGRF). Sequencing was performed on an Illumina NovaSeq 6000 at AGRF, generating 150bp paired-end reads.

Adapters, N content and low quality bases were trimmed from FASTQ files using fastq-mcf (version 1.05). Data was aligned to the b37 human genome reference from the BROAD which is based on GRCh37 using BWA MEM (version 0.7.17). The mapped data was sorted, and duplicates were marked using PICARD (version 2.17.3). Coverage estimation was performed by GATK3 DepthOfCoverage (version 3.8-1-0-gf15c1c3ef). Base Quality Score Recalibration was done using GATK4 BaseRecalibrator (version 4.0.10.1). Tumor purity was estimated using the cnv_facets implementation of the FACETS CN tool (version 0.6.1). To test for the presence of sample swaps all sequence data were assessed using HYSYS.

SNVs and INDELs in the 18 genes of interest (*BRCA1, BRCA2, RAD51C, RAD51B, RAD51D, BARD1, BRIP1, BRAF, PALB2, FANCM, TP53, PTEN, EGFR, CDK12, RB1, NF1, PI3KCA, CTNNB1;* Table S1C) were identified using VarDict (VarDictJava version 1.5.7) for germline variants, and somatic variants were identified using 4 tools: VarDict (VarDictJava version 1.5.7), VarScan2 (version 2.4.3), MuTect2 (version 4.0.11.0) and Strelka2 (version 2.9.9). Somatic variants were merged using GATK3 CombineVariants (version 3.8-1-0-gf15c1c3ef). Variants were annotated using Ensembl Variant Effect Predictor (version 92). Four tools were used to detect structural variants (SVs): GRIDSS (version 2.0.1), Manta (version 1.5.0), Smoove (version 0.2.2) and SvABA (version 134). High confidence SNV, INDEL and SVs were those identified by at least 2 variant calling tools. High confidence SNVs and INDELs were those that were represented at least once in each strand and were not in DUKE or DAC blacklist.

Correlations between genomic and anatomical distances

All disseminated tumor samples collected in this study were mapped to an anatomical location according to the interoperative mapping IMO 3x3 grid (Figure S1E). Due to detailed anatomical mapping of each sample collected, we investigated whether anatomical distances, derived using the IMO 3x3 grid, were associated with genomic distances within our cohort. Mantel tests were performed to identify correlations between sample-wise genomic and anatomical distances within each patient (n = 46). A one sample t-test was conducted on the absolute values of all correlation coefficients, with standard deviation of the absolute values of all correlation coefficients. A statistically significant difference was observed against the null distribution of correlation coefficient ($p = 4.99e-14$), indicating a significant shift toward a positive linear correlation between genomic and anatomical distances. p values of the Pearson's correlation coefficients are positively skewed, with p values concentrated on the lower end (median = 0.34, range = 0.01–1). The expected uniform distribution of p values was generated based on 10000 observations with a lower limit of 0 and upper limit of 1, which correspond to minimum and maximum p values respectively. One representative example case (T15-058) showing statistical significance (Mantel test, $p < 0.05$) is presented in Figure 5D, heatmaps of distance matrix derived from genomic distances (left) and anatomical distances (right).

Phenotypic apoptosis assays

Primary tumor cells were seeded in triplicate at a density of $1x10^4$ cells/well in 96 well plates and allowed to adhere for 48 h. Cells were treated with 25 μM cisplatin or media control followed by 24 h incubation at 37°C, 5% CO2. ApoTox-Glo Triplex Assay (Promega, US) was used to assess the apoptotic activity and cell viability within each well, measuring the active caspase- 3/7 and normalising the caspase activity to the viability of each sample measured, following the manufacture's protocol. In brief, GF-AFC reagent was added to each well and incubated for a minimum of 30 min at 37°C, the fluorescence signal was then measured with LUMIstar OPTIMA (BMG LabTech, UK), which was proportional to number of live cells. Then, Caspase-Glo 3/7 reagent was applied to each well for further incubation time (minimum 30 min) at RT and the relative luminescence unit (RLU) was measured on a LUMIstar OPTIMA as an indicator of caspase-3/7 activation and apoptosis. RLU was normalised to the relative cell viability fluorescence intensity, and calculated as a fold change to vehicle treated cells. Median absolute deviation (MAD) was used as a measure of variation of apoptosis induction of multiple tumor deposits for each patient. In total, >400 primary tumor cultures from n = 44 patients were assayed for apoptosis activity (n = 1 technical replicate per culture), as per other data analysis, data was excluded from further analysis for patients whose death was not due to disease progression, and cultures from two further cases discarded due to contamination.

IC_{50} assays

Primary tumor cells were seeded in triplicate at a density of $1x10^4$ cells/well in 96 well plates and allowed to adhere for 48 h. Half maximal inhibitory concentration (IC_{50}) was measured on primary cell cultures that were extracted and grown from tumor deposits from each patient. Cultures were treated with 2-fold serial dilutions from 100μM to 0μM of cisplatin for 72 h in standard 96-well microplates. An MTT assay was subsequently performed by adding MTT reagent (3-(4,5-dimethylthiazol-2-yl)-2,5-diphenyl tetrazolium bromide; Sigma-Aldrich) resuspended in PBS at 3 mg/mL. Three hours following the addition of MTT, stop solution (0.01% HCl and 10% SDS) was added and plates were incubated for a further 24 h. Absorbance at 570nm was measured the following day. Absorbance level was normalised against untreated wells.

Confirmation of primary tumor culture cell populations via Pax8 immunofluorescent staining

Primary tumor cells for cellularity determination were grown in parallel to the phenotypic assays performed for each case. Tumor cells from each deposit were seeded into 12 well plates at a density of $3x10^4$ cells/well and allowed to adhere for 48 h. Cells were then trypsinised with 1x Trypsin (Sigma-Aldrich, UK) diluted with 0.02% EDTA (Sigma-Aldrich, UK) and collected. Cells were centrifuged at 400xg for 5 min, supernatant discarded and resuspended in PBS. Cells were cytospun onto Superfrost Plus slides (VWR), air dried and stored at −20°C prior to staining. Cytospin slides were fixed with 4% paraformaldehyde (Santa Cruz Biotechnology, CA) at room temperature for 10 min. Slides were washed three times with 1xTris Buffered Saline (TBS), blocked and permeabilised using 10% Normal Goat Serum +0.5% Triton X- in TBS for 1 h at room temperature. Slides were incubated with Pax8 primary antibody (Abcam, UK), diluted at 1:50 in 10% Normal Goat Serum +0.1% Triton X- with 1xTBS at 4°C overnight, then washed three times with TBS. Slides were incubated with Alexa Fluor 488-conjugated anti-rabbit secondary antibodies diluted at 1:1000 in 10% Normal Goat Serum +0.1% Triton X- with 1xTBS for 2 h in dark conditions at 37°C. Slides were washed three times in 1xTBS and mounted using ProLong® Gold Antifade Mountant with DAPI (Life Technologies, UK), then left 24 h at room temperature in dark conditions to dry. Coverslips were then sealed and stored at 4°C prior to imaging. Imaging was performed using TCS SP5 confocal microscope (Leica, Germany).

A minimum of 10 random microscopy fields of each tumor sample were captured at 40× magnification. The total number of cells was acquired by counting all nuclei stained by DAPI, while the number of tumor cells was obtained by manually counting Pax8 immuno-positive cells. The percentage of tumor cells for each tumor deposit was determined and cultures with >70% tumor purity included for analysis.

Proteomic analysis

Primary tumor cells from multi-site tumor deposits were cultured as above and protein lysates were collected as per instructions for Reverse Phase Protein Array (MD Anderson Proteomics RPPA facility, US). Tumor lysates were serially diluted 2-fold for 5 dilutions, arrayed on nitrocellulose-coated slides and probed with 297 antibodies. Signals were amplified by a tyramide-based approach and visualised via a colorimetric reaction. Slides were scanned and spots quantified using the Array-Pro Analyzer software. Relative protein levels were determined by interpolation of each dilution curves from a standard curve constructed by the 'Super-Curve' package in R. All data points were normalised for protein loading and transformed to a linear value. Correction factors were implemented to correct for systematic error involved in protein loading. Samples with correction factor less than 0.25 or greater than 2.5 indicate the protein concentration is much lower or much higher than the other samples, hence are excluded from analysis. Correlations were performed for the CNEventDist by the proteomic distance, determined by the Euclidean distance between any pair of samples based on the protein log2 fold-change. Table S1D shows statistically significant positive correlations between proteomic and genomic heterogeneity.

Ki67 proliferative index staining and assessment

H&E and Ki67 stained slides were prepared for determination of tumor cell Ki67 proliferative index (PI) by manual microscopic assessments. Frozen tissue sections were collected from frozen tumors prior to DNA extraction for genomic profiling and were fixed in a 50:50 solution of acetone/methanol. The Ki67 antibody used was Abcam ab16667 (clone SP6) with automated immunohistochemistry performed on a Ventana platform with DAB as chromogen. Where possible Ki67 counts were carried out on approximately 1000 tumor cells per slide at ×20 magnification. In cases with less than 1000 tumor cells counts were performed on the available tumor cell population. As per standard practice for Ki67 assessments, faint but specific nuclear expression was scored as positive. For concordance of scoring methodology, the scorer (SMcQ) liaised with another colleague experienced in Ki67 assessments. In samples considered to be in a moderate/borderline Ki67 score (20–40% of Ki67 positive tumor cells within approximately 1000 tumor cells), then a second "hot-spot" count was also performed. Samples judged as inadequate for Ki67 assessments were recorded as such (lack of tumor or uninterpretable due to non-specific staining) and omitted from analysis. Three scoring categories were derived from the distribution of results from all assessed sections: low (0–20% Ki67 PI); moderate (21–60% Ki67 PI); and high (>60% Ki67 PI). Examples of staining for each category are shown in Figure S12A. Median absolute deviation (MAD) was used as a measure of variation of percentage Ki67 proliferative index of multiple tumor deposits for each patient. Stata SE 16.1 (StataCorp LLC, College Station, TX, USA) was used for the Ki67 analysis.

QUANTIFICATION AND STATISTICAL ANALYSIS

Statistical analysis

Statistical analyses were performed in R version 4.0.1. Survival analysis was performed using Cox proportional hazards regression model, implemented by the "survival" package. Mantel tests were implemented using the "ape" package. Empirical-Bayes moderated t-statistics for Linear regression models were obtained using the "limma" package. A p value below 0.05 was considered statistically significant and indicated with asterisk: $^{*}p < 0.05$, $^{**}p < 0.01$, $^{***}p < 0.001$. p values were adjusted for multiple testing using the false discovery rate (FDR) method. FDR-adjusted p values were reported as q values.

Cell Reports Medicine, Volume *4*

Supplemental information

Spatial and temporal intra-tumoral heterogeneity in advanced HGSOC: Implications for surgical and clinical outcomes

Paula Cunnea, Edward W. Curry, Elizabeth L. Christie, Katherine Nixon, Chun Hei Kwok, Ahwan Pandey, Ratri Wulandari, Kerstin Thol, Jennifer Ploski, Cristina Morera-Albert, Stephen McQuaid, Jingky Lozano-Kuehne, James J. Clark, Jonathan Krell, Euan A. Stronach, Iain A. McNeish, David D.L. Bowtell, and Christina Fotopoulou

Supplementary Information:

Figure S1: Survival analysis for patient cohort related to clinical parameters, clonal evolution, and relapse status.

Figure S2: Heterogeneous clustering patterns of tumor evolution for cases with relapse samples based on inter-deposit CNEvent genomic distances.

Figure S3: Variation in exposure to individual HGSOC copy number signature exposures grouped by relapse status and abdominal location, or clinical parameters.

Figure S4: Survival data for homologous recombination deficiency status, and poor prognosis biomarkers *CCNE1* and *MYC*

Figure S5: Quality control demonstrating copy number of genes associated with poor prognosis, aberrant cell fraction and ploidy for all tumor samples.

Figure S6: Phenotypic apoptosis, cisplatin IC_{50} and proliferative index data related to relapse status and patient outcomes for tumor samples in patient cohort.

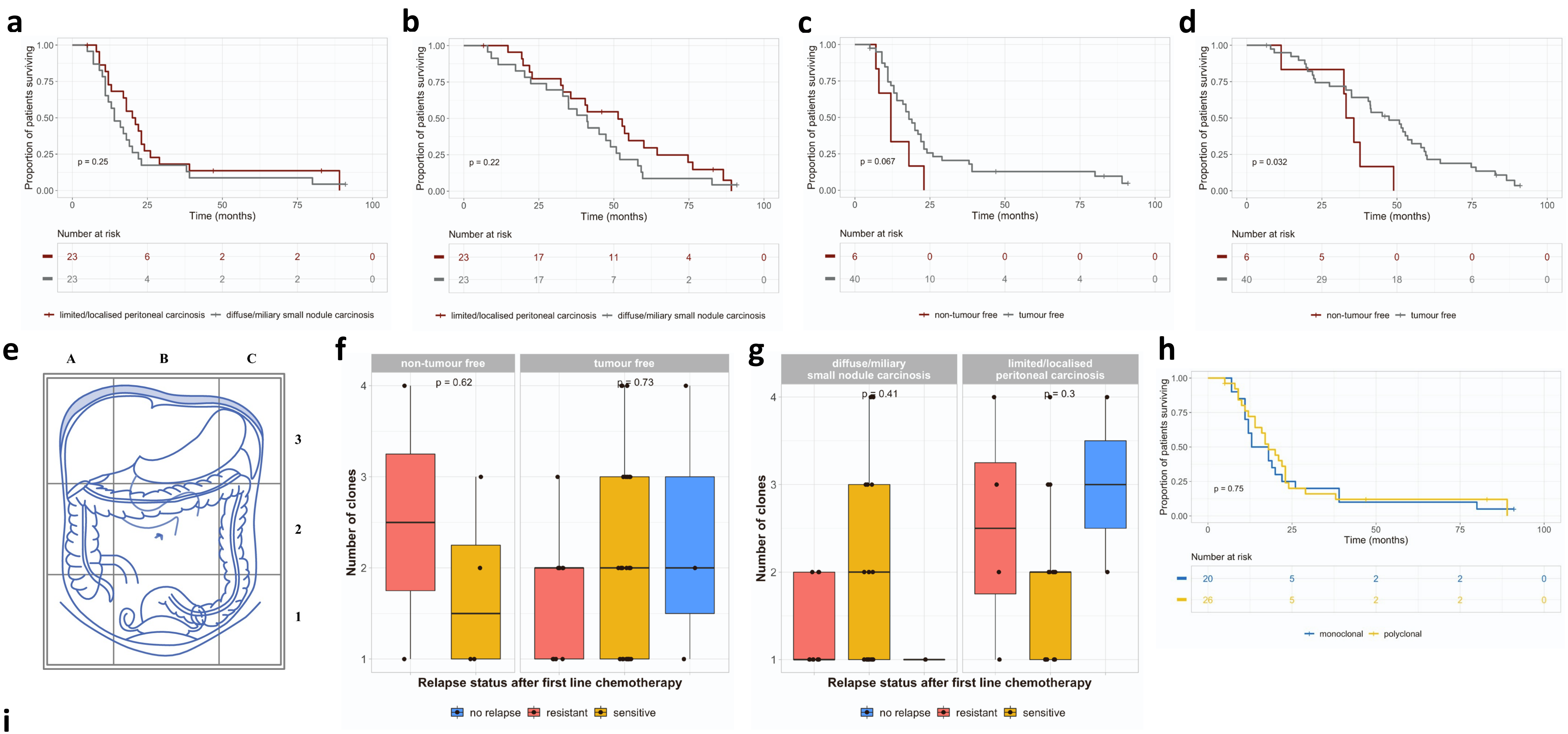

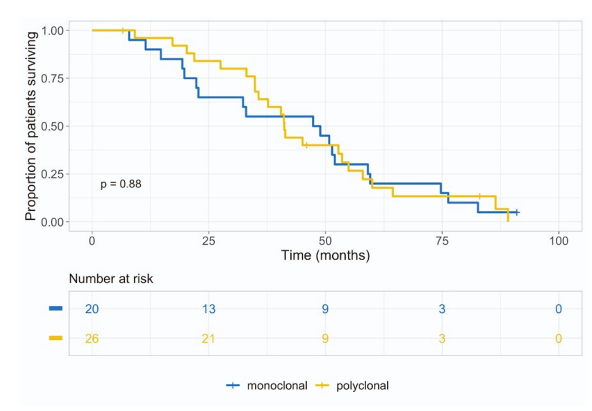

Figure S1 Survival analysis for patient cohort related to clinical parameters, clonal evolution, and relapse status.: a/b) Patient clinical outcomes by clinical presentation. Kaplan-Meier curve showing the association between disease status (limited/localized peritoneal carcinosis; diffuse/military small nodule carcinosis) and progression free survival (**a;** p = 0.25) and overall survival (**b;** p = 0.22) (n = 46). **c/d) Patient clinical outcomes by presence of residual disease following primary debulking surgery (PDS).** Kaplan-Meier curve showing the significant association between tumor-free status (n=40) vs non-tumor free (n=6) and progression free survival (**c;** p = 0.067) and overall survival (**d;** p = 0.032). **e) Intra-operative mapping tool for ovarian cancer (IMO). f) Variation in the number of clones by presence of residual disease post PDS.** Box plot showing the number of clones in primary deposits from patients (n = 46), grouped by presence of residual disease

following PDS (tumor-free versus non-tumor free status) and the relapse status after first line chemotherapy. Wilcoxon signed-rank test was performed across patients of different relapse status within residual disease groups. No statistically significant differences were observed**. g) Variation in the number of clones by tumor dissemination patterns.** Box plot showing the number of clones in primary deposits from patients (n = 46), grouped by the dissemination pattern (limited/localized peritoneal carcinosis; diffuse/military small nodule carcinosis) and relapse status after first line chemotherapy. Wilcoxon signed-rank test was performed across patients of different relapse status within each dissemination pattern group. **h/i) Patient clinical outcome by primary tumor clonal diversity.** Kaplan-Meier plots showing association between number of clones in primary tumor deposits (monoclonal versus polyclonal) and progression-free survival (**c:** PFS, p = 0.75) and overall survival (**d:** OS, p = 0.88). Related to Figure 2.

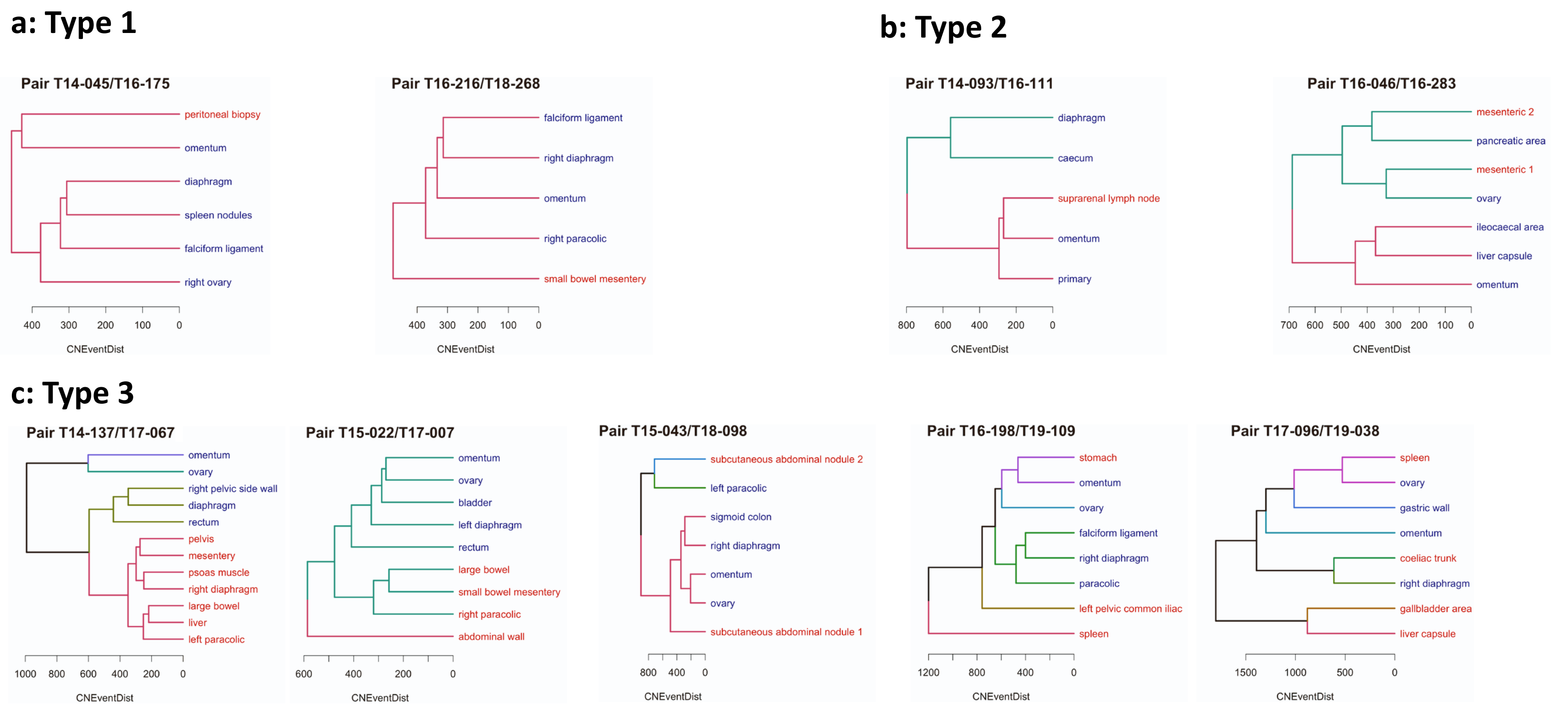

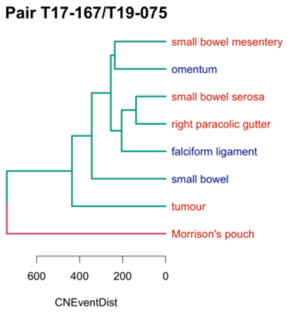

Figure S2: a-c: Heterogeneous clustering patterns of tumor evolution for cases with relapse samples based on inter-deposit CNEvent genomic distances. Companion to Fig.2g, showing the remaining 7 cases with relapse samples categorized into Types 1 (**a**), 2 (**b**) and 3 (**c**) according to the pattern of clonal evolution. Tumors collected at time of primary surgery are labelled in blue, tumors collected at relapse are labelled in red, the color of the dendrogram branches represents different clones.

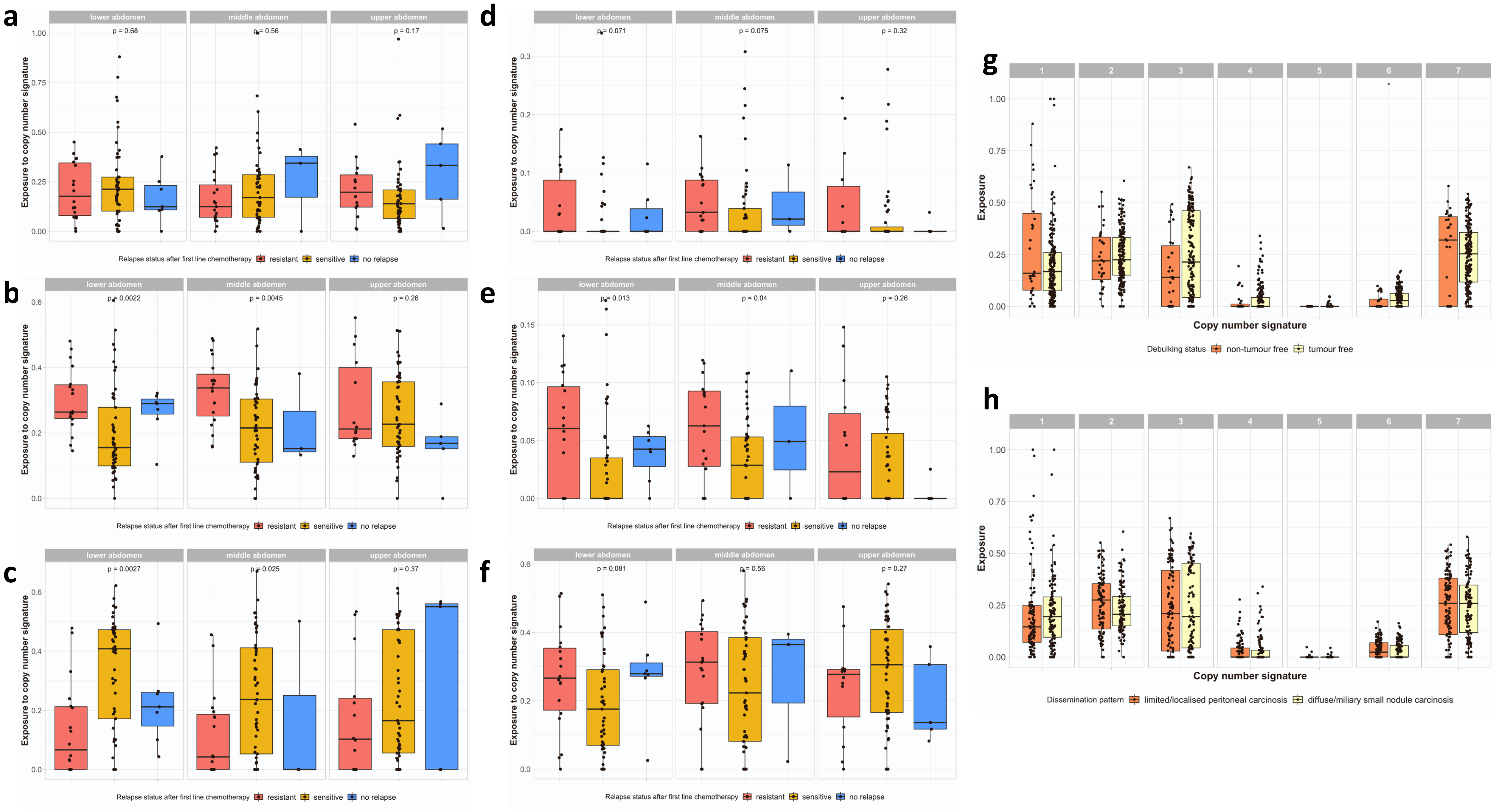

a
lower abdomen
middle abdomen
upper abdomen
p = 0.68
p = 0.56
p = 0.17
Exposure to copy number signature
Relapse status after first line chemotherapy
resistant
sensitive
no relapse
b
p = 0.0022
p = 0.0045
p = 0.26
c
p = 0.0027
p = 0.025
p = 0.37
d
p = 0.071
p = 0.075
p = 0.32
e
p = 0.013
p = 0.04
p = 0.26
f
p = 0.081
p = 0.56
p = 0.27
g
Exposure
Copy number signature
Debulking status
non-tumour free
tumour free
h
Dissemination pattern
limited/localised peritoneal carcinosis
diffuse/miliary small nodule carcinosis

Figure S3 Variation in exposure to individual HGSOC copy number signature exposures grouped by relapse status and abdominal location, or clinical parameters: a-f) Variation in exposure to individual HGSOC copy number signature exposures grouped by relapse status and abdominal location (upper (IMO A3-C3), middle (IMO A2-C2) and lower (IMO A1-C1) abdomen). a: Signature 1, **b**: Signature 2, **c**: Signature 3, **d**: Signature 4, **e**: Signature 6, **f**: Signature 7. Statistically significant differences in relapse groups compared to sensitive/no relapse groups were observed in the poor prognosis Signatures 2 and 6 for tumors located in the lower (S2: $p = 0.0022$; S6: $p = 0.013$) and middle (S2: $p = 0.0045$; S6: $p = 0.04$) abdominal areas, but not the upper abdomen (Kruskal-Wallis test). Signature 3 displays significant elevated signature scores in the sensitive/no relapse groups compared to the resistant group in the lower abdomen ($p = 0.027$) and middle abdomen ($p=0.025$). There was little to no representation of Signature 5, related to subclonal catastrophic chromothriptic-like events through unknown mechanisms, in any of the tumors profiled and therefore a graph for signature 5 was not plotted. **g) Variation in exposure to HGSOC copy number signatures grouped by debulking status.** Box plot showing the exposure to 7 copy number signatures of HGSOC for 215 samples from 46 patients, grouped by debulking status of primary surgery (tumor free vs non-tumor free). Wilcoxon signed-rank test was performed across samples from patients of different debulking status within each copy number signature group. Statistical significance was observed for Signature 6 ($p = 0.049$). **h) Variation in exposure to HGSOC copy number signatures by dissemination patterns.** Box plot as in (**e**) grouped by dissemination pattern of tumors observed in patients. Statistical test as (**g**). Related to Figure 3.

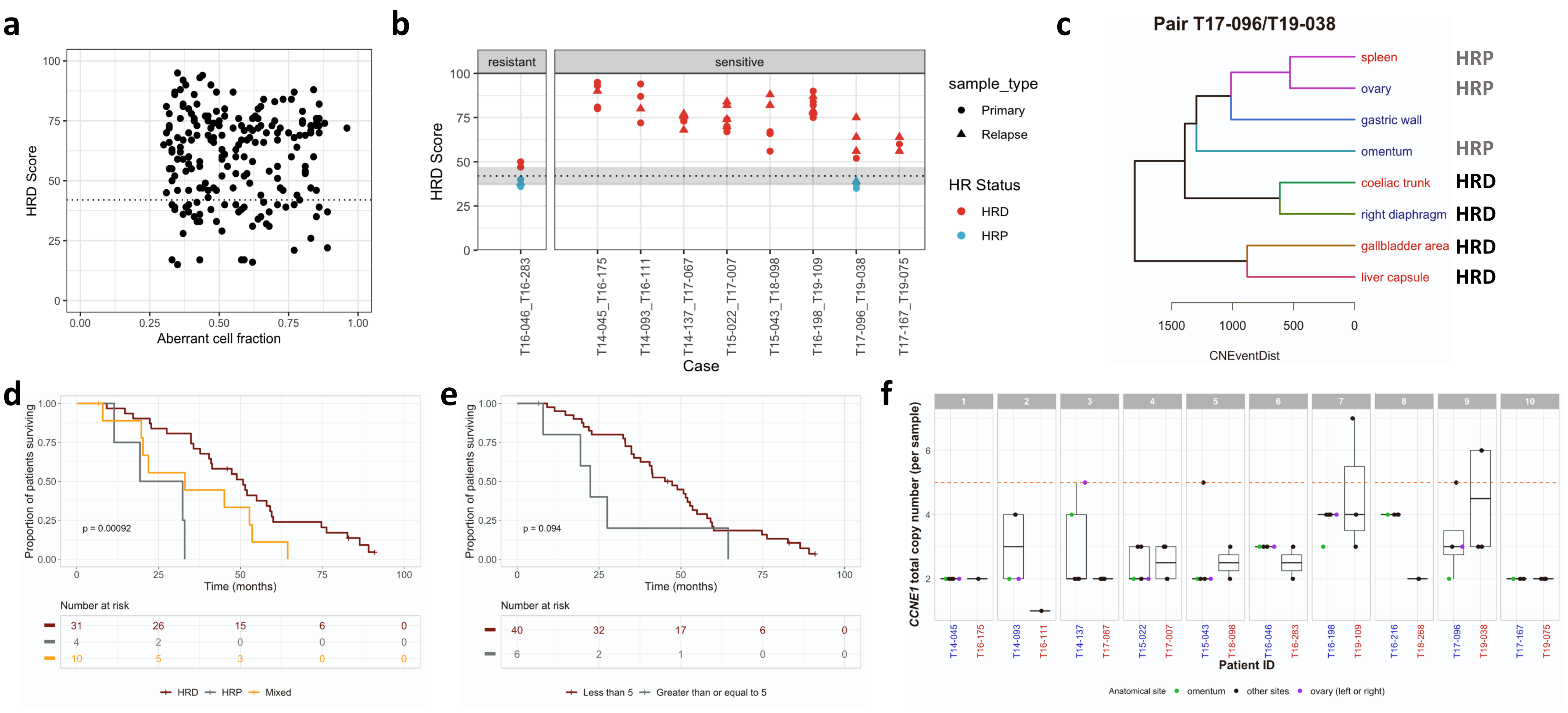

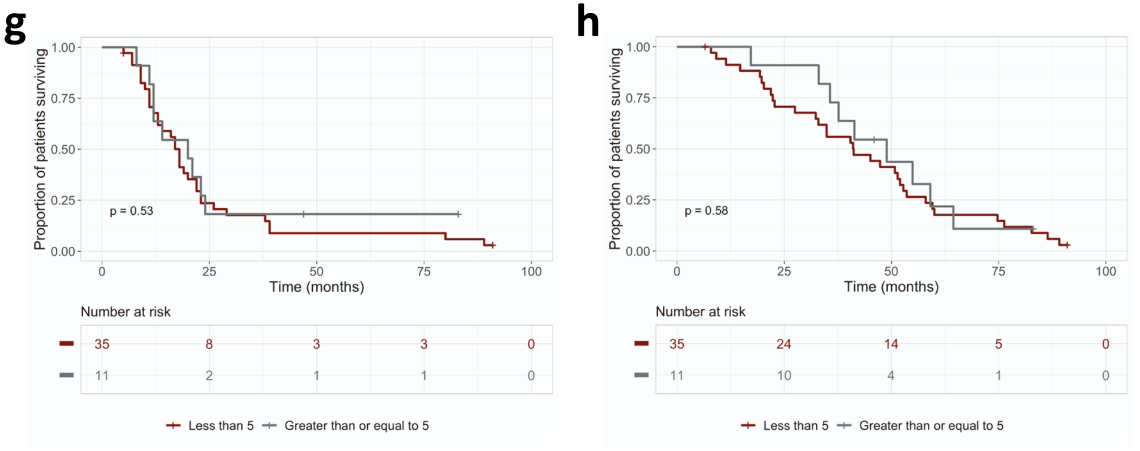

Figure S4 Survival data for homologous recombination deficiency status, and poor prognosis biomarkers CCNE1 and MYC: a) Quality control demonstrating aberrant cell fraction in tumors profiled for HR score. Aberrant cell fraction was estimated for all tumors in the cohort, and those with an ASCAT aberrant cell fraction of <0.3 were excluded due to low tumor purity. Samples also with an ASCAT aberrant cell fraction of 1 and an HRD-LOH, LST and telomeric AI score of 0 were also excluded. Samples were classified as HR deficient (HRD) if the HR Sum score was >42 (dotted line). **b) HR profiles observed in cases with relapse tumor samples.** HR scores are shown for each tumor for 9 patients who had

tumors collected at time of relapse. For two of the patients, mixed HR status was observed within both the primary and relapse samples. **c) Relapse tumors cluster with primary tumors according to HR status.** For patient T17-096, we examined the tumor evolution pattern from primary to relapse as demonstrated by dendrogram derived from tumor genomic distances. The spleen tumor collected at relapse, categorized as HR proficient, clustered with the tumors collected at primary surgery that were also classified as HR proficient. The other relapse samples: coeliac trunk, gall bladder and liver capsule clustered with the diaphragm sample from primary surgery, all classified as HR deficient. **d) Clinical implication of HR status**. Kaplan-Meier curve showing the association between the 3 different HR categories (all tumors within a patient had a HRP profile (HRP), all tumors had a HRD profile (HRD) or if there was a mix of HRP and HRD profiles within a patient's tumors (Mixed)) and overall survival (OS, p = 0.00092). **e) Clinical implication of CCNE1 amplification**. Kaplan-Meier curve showing the association between the mean *CCNE1* total copy number across multiple deposits within a patient and overall survival (OS), split by a mean *CCNE1* copy number of greater or less than 5 copies (OS, p = 0.091). **f) Intra-tumor heterogeneity of *CCNE1* copy number in cases with relapse samples**. Paired box plot showing *CCNE1* total copy number per sample across patients (n = 10), grouped by collection time point and points colored by tumor anatomical site. Blue text indicates tumor deposits collected from the primary surgery, while red text indicates tumor deposits collected at relapse. **g/h) Clinical association of *MYC* amplification**. Kaplan-Meier curve showing the association between the mean *MYC* total copy number across multiple deposits within a patient and progression free survival (**g:** PFS, p = 0.53) and overall survival (**h:** OS, p = 0.58), with patients stratified into 2 groups based on the mean *MYC* copy-gain of less than or great than or equal to 5 copies. Related to Figure 4.

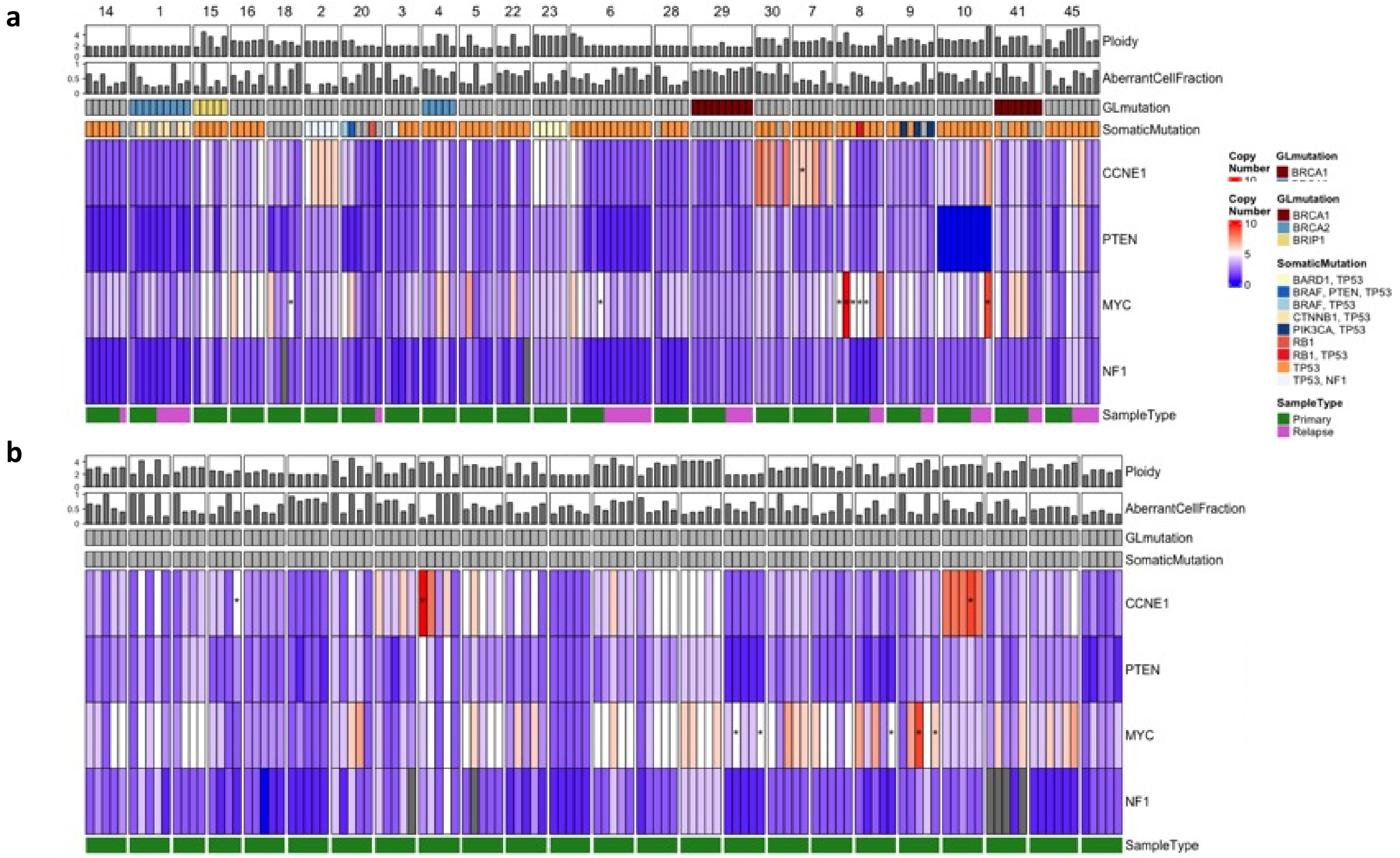

Figure S5: Quality control demonstrating copy number of genes associated with poor prognosis, aberrant cell fraction and ploidy for all tumor samples. a) Heatmap depicting the *CCNE1, MYC, PTEN* and *NF1* total CN in all tumors profiled (n=259), annotated by tumor ploidy, aberrant cell fraction, and sample collection time. Samples from the same case are grouped together. Gene amplification as defined by COSMIC (ploidy ≤2.7 and total CN ≥5 or ploidy >2.7 and total CN ≥9) is highlighted using asterisks*. Somatic and germline mutational data available for 21 patients including matched relapse is displayed according to data legend. **b)** Heatmap as in **(a)** depicting *CCNE1, MYC, PTEN* and *NF1* total CN in tumors, without germline or somatic mutational data. See also Figure 4d; Supplementary File 1 - Table S1f.

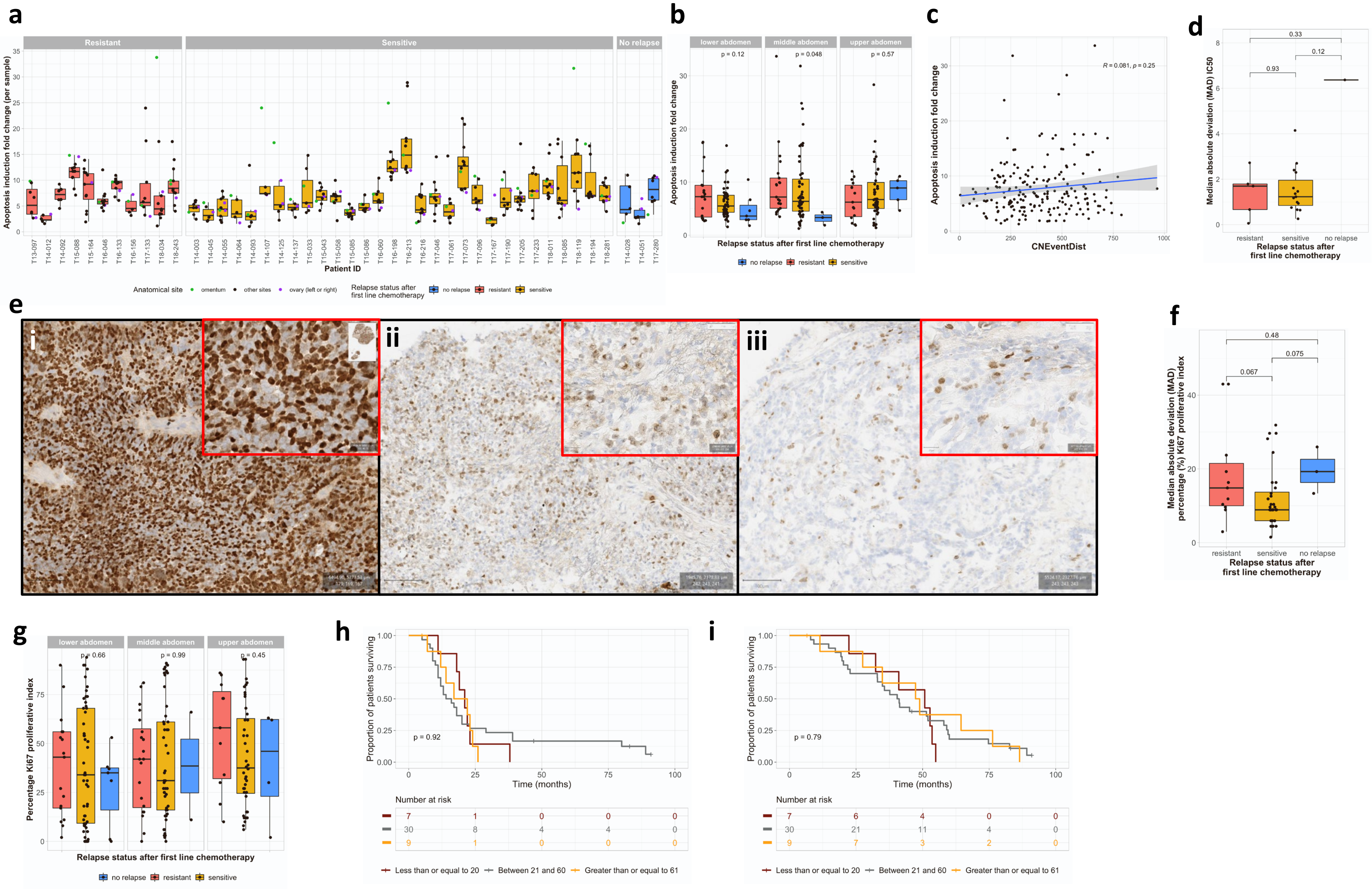

a
Resistant
Sensitive
No relapse
Apoptosis induction fold change (per sample)
Patient ID
Anatomical site
omentum
other sites
ovary (left or right)
Relapse status after first line chemotherapy
no relapse
resistant
sensitive
b
lower abdomen
middle abdomen
upper abdomen
p = 0.12
p = 0.048
p = 0.57
Apoptosis induction fold change
Relapse status after first line chemotherapy
c
R = 0.081, p = 0.25
CNEventDist
d
Median absolute deviation (MAD) IC50
0.33
0.12
0.93
resistant
sensitive
no relapse
Relapse status after first line chemotherapy
e
i
ii
iii
f
Median absolute deviation (MAD) percentage (%) Ki67 proliferative index
0.48
0.075
0.067
Relapse status after first line chemotherapy
g
Percentage Ki67 proliferative index
p = 0.66
p = 0.99
p = 0.45
Relapse status after first line chemotherapy
h
Proportion of patients surviving
p = 0.92
Time (months)
Number at risk
Less than or equal to 20
Between 21 and 60
Greater than or equal to 61
i
p = 0.79

Figure S6 Phenotypic apoptosis, cisplatin IC50 and proliferative index data related to relapse status and patient outcomes for tumor samples in patient cohort: a) *In vitro* apoptosis levels following platinum chemotherapy treatment by patient. Related to Figure 5. Box plot of phenotypic apoptosis induction of tumor cultures derived from each patient according to relapse status. Primary 2D-tumor cell cultures were established from disseminated tumors (mean=9, range 4-15) and treated in triplicate with cisplatin to evaluate *in vitro* apoptotic responses to platinum chemotherapy, data was normalized to viability and a fold change of apoptosis induction was determined compared to vehicle control for each tumor, n=1 technical replicates. **b) *In vitro* apoptosis levels following platinum chemotherapy treatment by abdominal location.** Functional heterogeneity of primary tumor cells is demonstrated using a box plot showing the apoptosis induction fold change of primary cell cultures that were extracted and grown from tumor deposits by abdominal location (lower, middle, or upper), grouped by relapse status. There is a trend towards higher apoptosis induction in the resistant group in the lower ($p = 0.12$) and middle ($p = 0.048$) abdominal areas (Kruskal-Wallis test). **c) Association of apoptotic response with genomic distance.** Non-significant correlation between genomic distance (CNEventDist) and apoptotic induction for each tumor per patient (r=0.081, p=0.25). **d) Functional heterogeneity of primary tumor cells.** Box plot showing the median absolute deviation (MAD) of sample-wise IC50 for cisplatin from primary cell cultures that were grown from tumor deposits from a subset of patients (n=21), grouped by relapse status. Kruskal-Wallis test, p=0.2537, related to Table S1i. **e) Percentage Ki67 proliferative index examples of staining categories.** Related to Figure 5e. Representative images of each of the categories: **i** High Ki67, **ii** Moderate Ki67, **iii** Low Ki67. Main images are at x4 magnification and inserts at x20 magnification. Scale bar at 100 µm. **f) Heterogeneity in Ki67 proliferative index of primary tumors.** Box plot showing the median absolute deviation (MAD) of sample-wise Ki67 proliferative index of tumor deposits from patients (n = 46 patients), grouped by relapse status (Kruskal-Wallis test, p = 0.05278). **g) Distribution of Ki67 proliferative index scores across different abdominal areas.** Percentage Ki67 proliferative index was grouped by relapse status and plotted according to abdominal location (lower, middle, upper). No statistically significant differences were observed across the 3 abdominal areas and relapse groups (Kruskal-Wallis test). **h/i) Clinical association of Ki67 proliferative index**. Kaplan-Meier curve showing the association between the mean Ki67 PI across multiple deposits within a patient and progression free survival (PFS, p = 0.92) and overall survival (OS, p = 0.79), with positive Ki67 proliferative index staining stratified into 3 categories: low (<20%), moderate (21-60%) and high (>60%) across the cohort (n=46)
